# Clustering of health risk behaviors among adolescents in Kilifi, Kenya, a rural Sub-Saharan African setting

**DOI:** 10.1371/journal.pone.0242186

**Published:** 2020-11-12

**Authors:** Derrick Ssewanyana, Amina Abubakar, Charles R. J. C. Newton, Mark Otiende, George Mochamah, Christopher Nyundo, David Walumbe, Gideon Nyutu, David Amadi, Aoife M. Doyle, David A. Ross, Amek Nyaguara, Thomas N. Williams, Evasius Bauni

**Affiliations:** 1 Centre for Geographic Medicine Research Coast, Kenya Medical Research Institute (KEMRI), Kilifi, Kenya; 2 Utrecht Centre for Child and Adolescent Studies, Utrecht University, Utrecht, The Netherlands; 3 Institute for Human Development, Aga Khan University, Nairobi, Kenya; 4 Department of Psychiatry, Warneford Hospital, University of Oxford, Oxford, United Kingdom; 5 INDEPTH (International Network for field sites with continuous Demographic Evaluation of Populations and Their Health in developing countries), East Legon, Accra, Ghana; 6 London School of Hygiene & Tropical Medicine, Bloomsbury, London, United Kingdom; 7 Department of Medicine, Imperial College, South Kensington Campus, London, United Kingdom; University of the Witwatersrand, SOUTH AFRICA

## Abstract

**Background:**

Adolescents tend to experience heightened vulnerability to risky and reckless behavior. Adolescents living in rural settings may often experience poverty and a host of risk factors which can increase their vulnerability to various forms of health risk behavior (HRB). Understanding HRB clustering and its underlying factors among adolescents is important for intervention planning and health promotion. This study examines the co-occurrence of injury and violence, substance use, hygiene, physical activity, and diet-related risk behaviors among adolescents in a rural setting on the Kenyan coast. Specifically, the study objectives were to identify clusters of HRB; based on five categories of health risk behavior, and to identify the factors associated with HRB clustering.

**Methods:**

A cross-sectional survey was conducted of a random sample of 1060 adolescents aged 13–19 years living within the area covered by the Kilifi Health and Demographic Surveillance System. Participants completed a questionnaire on health behaviors which was administered via an Audio Computer-Assisted Self–Interview. Latent class analysis on 13 behavioral factors (injury and violence, hygiene, alcohol tobacco and drug use, physical activity, and dietary related behavior) was used to identify clustering and stepwise ordinal logistic regression with nonparametric bootstrapping identified the factors associated with clustering. The variables of age, sex, education level, school attendance, mental health, form of residence and level of parental monitoring were included in the initial stepwise regression model.

**Results:**

We identified 3 behavioral clusters (Cluster 1: *Low-risk takers (22*.*9%);* Cluster 2: *Moderate risk-takers (67*.*8%);* Cluster 3: *High risk-takers (9*.*3%)*). Relative to the cluster 1, membership of higher risk clusters (i.e. moderate or high risk-takers) was strongly associated with older age (p<0.001), being male (p<0.001), depressive symptoms (p = 0.005), school non-attendance (p = 0.001) and a low level of parental monitoring (p<0.001).

**Conclusion:**

There is clustering of health risk behaviors that underlies communicable and non-communicable diseases among adolescents in rural coastal Kenya. This suggests the urgent need for targeted multi-component health behavior interventions that simultaneously address all aspects of adolescent health and well-being, including the mental health needs of adolescents.

## Introduction

Adolescents (10–19 years) have a high propensity for risk-taking which can lead to health risk behavior (HRB) clustering or co-occurrence of multiple forms of risky behavior [1, 2]. HRB are specific forms of behavior associated with increased susceptibility to a specific disease or ill health as shown in epidemiological or social data. Examples of HRB include behavior that contributes to unintentional injury or violence, unhealthy dietary habits, inadequate physical activity, sexual behaviors contributing to unintended pregnancy and sexually transmitted diseases, alcohol, tobacco and drug use [3, 4]. Clustering is when the observed proportion of a combination of risk factors exceeds its expected proportion [5]. The mechanisms underlying HRB clustering are poorly understood [2]. Although there has been growing interest in HRB clustering from other parts of the world such as Europe and North America [6–9], there is a dearth of research on this topic among adolescents from sub-Saharan Africa (SSA), which has the largest proportion of young people [10]. Besides, existing research suggests that a multiple-risk-behavior approach is often not reflected in the formulation of policies and interventions to reduce adolescent HRB [7]. Therefore, the need for more holistic and viable HRB assessment, such as clustering of HRB during adolescence has been highlighted as crucial for effective planning of adolescent health promotion interventions [2, 11].

Some of the early literature on adolescent behavior, for example by Jessor, describes a tendency for covariation among risk behaviors which can result in a ‘risk behavior syndrome’ [12, 13]. Jessor’s problem-behavior theory posits that three major systems comprise explanatory variables for problem-behavior and that proneness to such behavior is explained by the extent of balance between the instigations and controls within each system. These systems are: perceived-environment system (consisting of proximal and distal variables of social controls, support and models); personality system (comprising interrelated socio-cognitive variables such as beliefs, expectation, and attitudes); and the behavior system (consisting of both problem behavior such as HRB, and conventional behavior like religiosity and academic involvement) [12]. Specifically within the behavior system, it is theorized that involvement in one problem behavior increases the likelihood of involvement in the other forms of problem behavior. This covariation among risk behavior is attributed to the shared social ecology by youths which may offer socially organized opportunities and normative expectations to learn and engage in multiple forms of risky behavior. Covariation may also occur due to a similarity in psychological meaning and purpose attached to different forms of risk behavior [14, 15]. For example, young people may engage in substance use and risky sexual behavior as a way of affirming their independence and gaining acceptance in their peer networks [12, 13]. Jessor deduces that a consideration of covariation (clustering) of multiple risky behaviors directs attention to influencing the adolescent’s lifestyle as a whole rather than focusing on specific behavior.

Substance use, behavior resulting in injury, poor personal hygiene, poor diet, and low levels of physical activity make significant contributions to the burden of morbidity and mortality in adolescence [16, 17]. The clustering of these and other forms of HRB may aggravate poor educational attainment, poor social and health outcomes during adolescence, as well as shape an unhealthy or socially problematic lifestyle during adulthood [18]. Harmful use of alcohol, unhealthy diet, tobacco use and physical inactivity are linked to an increased risk of dying from non-communicable diseases (NCDs) like diabetes, cardiovascular diseases, cancers and respiratory conditions [19, 20]. These forms of behavior are found to cluster among adolescents [21–23] and this could potentially exacerbate risk to NCDs. A recent survey of 2540 school adolescents in Seychelles [21] for example, reported a high prevalence of inadequate fruit and vegetable consumption (61%), physical inactivity (83%), high consumption of soft drinks (68%), current alcohol use (48%), and current tobacco use (23%). Moreover, the co-occurrence of 3 or more forms of risky behavior linked to NCDs was 81% within this sample [21]. Another survey among 8 African countries (Kenya, Namibia, Morocco, Swaziland, Tanzania, Uganda, Zambia and Zimbabwe) found high prevalence (25%-63%) of bullying (on at least 1 day within the past 30 days) among the adolescents [24]. This study also found that adolescents’ exposure to bullying was strongly significantly associated with their current cigarette use, current alcohol use, lifetime drug use, and multiple sexual partnerships [24]. These findings on HRB covariation were also corroborated by those from another multi-country survey of 7 African states which reported a high (15.5%) prevalence of early initiation of tobacco smoking among adolescents and its significant association with alcohol and other drug use, unintentional injuries and violencer [25]. In both multi-country surveys, only the associations between the main HRB of interest (bullying [24], early initiation of tobacco smoking [25]) and isolated forms of HRB were investigated as opposed to the approach of clustering of multiple forms of HRB.

Behaviors such as sub-optimal oral hygiene, poor handwashing practices and inadequate body hygiene have been linked to increased risk for communicable diseases like enteric infections [26], acute respiratory infections [27], and trachoma [28]. The clustering of these forms of behavior is also increasingly documented among adolescents [29–31]. For instance, a survey conducted among adolescents from 9 African countries reported sub-optimal tooth brushing among 22% and only 58% of the sample reported regular handwashing after using the toilet [31]. Some studies among adolescents have also reported an overlap between behaviors linked to communicable diseases and NCDs [32, 33]. This behavioral overlap (i.e. of both forms of behavior linked to communicable diseases and NCDs) may partly explain the double burden of NCDs and communicable diseases currently faced in low resource settings like SSA [34]. In general, the existing body of research indicates an urgent need for more holistic approaches to HRB research among adolescents as this could fill knowledge gaps on how to appropriately design and implement HRB intervention programs.

With up to a third of all deaths in Kenya attributable to NCDs, there is evidence of a growing burden of NCDs in this region, just like in many other low- and middle-income countries (LMICs) [35]. Studies conducted among the Kenyan adolescent sub-population highlight a high burden (61% among girls and 36% among boys) of sub-optimal engagement in physical activity [36]; early onset (10 years) and general increase in alcohol, tobacco and other drug use [37]; sub-optimal hygiene practices [31, 38]; and a high burden of bullying [24], physical and sexual violence (4% - 48%) [39]. However, it is common that individual HRB is mostly reported in isolation within the Kenyan context thus a missed opportunity to identify the most vulnerable adolescents and the most crucial cross-cutting factors to target when simultaneously intervening multiple forms of HRB. Noteworthy, a holistic approach to HRB reporting such as HRB clustering, aligns with the strategic objective of promoting lifestyle and implementing interventions to reduce priority modifiable risk factors (physical inactivity, unhealthy diet, harmful use of alcohol and tobacco use, and violence and injuries) as stipulated in Kenya’s national strategy for the prevention and control of non-communicable diseases [40, 41]. A better understanding of patterns of HRB co-occurrence will inform interventions and adolescent health promotion policies.

Variations in the sources of vulnerability to HRB clustering may exist across Kenyan counties. Adolescents who reside in poorer settings such as Kilifi County may experience disproportionately poorer behavioral health. Indeed, previous studies have reported abject poverty (about 58% of Kilifi residents live below the poverty line [42]), poor educational outcomes (high drop-out and poor transition between grades), detrimental cultural practices, and poor accessibility to good quality adolescent-friendly services as some of the sources of vulnerability among youths in Kilifi county [43–45]. These numerous sources of vulnerability are likely to predispose adolescents in this rural setting to multiple forms of HRB and injustices. Thus, there is an urgent necessity for more comprehensive research with a multiple-risk-behavior approach to guide a more cohesive and efficient approach to adolescent risk in this context. The objectives of this study, which utilizes data collected in a Kilifi county which is a rural setting at the Kenyan coast, were: (i) to identify clusters of health risk behavior among adolescents living in Kilifi based on 5 behavioral categories (injury or violence, substance use, hygiene, physical activity, and dietary behavior); and (ii) to identify the factors associated with health risk behavior clustering.

## Materials and methods

### Study design

This was a cross-sectional survey conducted between August and December 2014, among young people aged 13–24 years who were residents of the area covered by the Kilifi Health and Demographic Surveillance System (KHDSS). Using the KHDSS population register, stratified random sampling based on age and sex was utilized to draw a sample of 2072 study participants who were then contacted by the study team through household visits. Of the 2072 potential study participants, consent and/or assent were obtained from 1524 (73.6%) aged 13–24 years. Those whose consent and/or assent were not obtained was due to their decline to participate in the study (3.0% of the total sample), having trans-migrated or out-migrated from the KHDSS (11.7%), absence from home during the household visit (8.1%), and other reasons such as procrastination by the participant (3.6%). All the survey questions were administered via an Audio Computer-Assisted Self–Interview (ACASI) programmed in any of the 3 languages of English, Swahili and Giryama. The data were collected during household visits prior to which a participant would be instructed in detail on how to use the ACASI and left to complete the interview in private on a touch-screen laptop with a pair of headphones.

### Study setting

The residents of the KHDSS mainly belong to the Mijikenda ethnic group, and their native language is one of the Mijikenda languages (commonly Giriama) and Swahili. It is estimated that nearly 58% of the residents of Kilifi County live below the poverty line and a third have not attained any formal education [42, 46]. By 2016, about 61% of the 1.4 million residents of Kilifi County were rural dwellers. About 22% of the residents were adolescents. Local home business and subsistence farming are the major sources of livelihood, however, the transport industry (such as motorcycle taxi and tuk-tuk) has become a significant source of livelihood for many unemployed youths [43].

### Participants

The current study focuses on the 1060 adolescents aged 13–19 years (70% of the total 1524 13-24-year-olds who participated in the survey). The focus on the 13–19 year age-group was to ensure that this specific study succinctly focuses on the adolescence developmental period. Written parental or guardian consent as well as adolescents’ assent were obtained from participants aged 13–17 years. Written consent was directly sought from adolescents aged 18–19 years and those who were less than 18 years but were married (4%). Ethical approval was obtained from the Kenya Medical Research Institute National Scientific and Ethical Committee (Number 2823) and the Ethics Committee of the London School of Hygiene and Tropical Medicine.

### Measures

Five behavioral categories of injury or violence-related behavior, substance use, hygiene behavior, physical activity, and dietary behavior were utilized in this study. All items/questions used to assess these behavioral factors (see S1 File) were borrowed from the Global School–Based Student Health Survey (GSHS) 2013 core questionnaire modules [47].

### Injury or violence-related behavior

Participants were asked about the number of times they were seriously injured in the past 12 months (response options ranged from ‘0 times’ to ‘12 or more times’) and in the analysis the data was categorized into 3 levels of “Never”, “Once” and “More than once” for easier interpretation of the results.

Bullying or victimization was assessed by asking participants how many days they were bullied during the past 30 days (response options ranged from ‘0 days’ to ‘all the 30 days’). In our analysis, this item was re-categorized to “0 days”, “1–5 days” and “6 or more days” for simplicity.

#### Substance use

Cigarette smoking was assessed by asking participants how old they were when they first tried smoking a cigarette (responses were categorized to “Never smoked”, “13 years or less” and “14 years and above” so as to capture early onset of smoking behavior).

Participants were also asked on how many days they had smoked during the past 30 days (categorized to “No” if the response was ‘0 days’ and “Yes” if the participant had smoked during any of the 30 days so as to capture current/recent smoking behavior).

Alcohol consumption was assessed by asking participants how old they were when they first had a drink of alcohol other than a few sips (categorized to “Never drank alcohol” (or only a few sips), “13 years or less” and “14 years and above” in order to capture early onset of alcohol consumption). The participants were also asked if they had at least one drink containing alcohol during the past 30 days (categorized to “No” if the response was ‘0 days’ and “Yes” if the participant had drunk at least one alcoholic drink during any of the 30 days so as to capture current/recent alcohol consumption).

Lifetime use of marijuana was probed by asking how many times the respondent had used marijuana in their lifetime (categorized to “No” if the response was ‘0 times’ and “Yes” if the participant had ever used marijuana).

In the survey questions, locally relevant names and examples of substances were used.

#### Hygiene behavior

Oral hygiene was assessed by asking participants how many times per day they cleaned or brushed their teeth in the past 30 days (response options ranged from ‘did not clean or brush teeth’ to ‘4 or more times per day’). The responses were re-categorized to “Poor” if the response was ‘did not clean or brush teeth’ or *‘*did so less than daily’ and to “Adequate” if the response ranged from ‘once a day’ to ‘4 or more times per day’.

The participants were asked how often they washed their hands after using the toilet or latrine in the past 30 days (response options ranged from ‘never’ to ‘always’). We re-categorized responses to: “Poor” if the response was ‘never’ or ‘rarely’; to “Sometimes” if the response was ‘sometimes’; and to “Adequate” if the response was ‘most of the time’ or ‘always’.

#### Physical activity

Participants were asked how many days they were physically active for a total of at least 60 minutes per day during the past 7 days. Their responses were categorized into “0 days”, “1–4 days” and “5–7 days” for easier interpretation of the results. They were also asked about the time they spent on a typical or usual day sitting and watching television, chatting with friends or doing other sitting activities (response options ranged from ‘less than 1 hour per day’ to ‘more than 8 hours per day’) and we reclassified their response options to “4 hour or less” and “5 or more hours” [48].

#### Dietary behavior

Daily fruit consumption was assessed by asking respondents how many times per day they usually ate fruits during the past 30 days (response options ranged from ‘did not eat fruits’ to ‘5 or more times per day’). We re-categorized the response options to “0 or less than 1 time”, “1–2 times”, and “3 times or more”.

Participants were also asked how many days they ate food from a fast-food restaurant during the past 7 days. Their responses were re-categorized to “0 days”, “1–4 days” and “5–7 days”. While asking both questions, locally relevant examples of fruits and fast foods were used.

#### Other variables (social demographics and co-variates)

We hypothesized that the demographic characteristics of a respondent, the degree to which their parents/guardians monitored their behavior [49], and symptoms of depression in the past two weeks might be associated with risk group membership [50].

Respondents’ age, sex, area of residence, and school attendance were ascertained during the survey. Participants’ age was generated by computing the difference between the date of survey completion by the participant and their date of birth verified from written records such as birth certificates or healthcare documents. School attendance was assessed by asking the participants if they were currently attending school. The response options were “No” and “Yes”. Upon completion of the consenting process at the participant’s household, their area of residence was selected by the research assistant from the computer’s uploaded list of sub-locations within the KHDSS.

Parental monitoring during past 30 days was assessed by asking the respondents how often their parents or guardians knew what they did during their free time in the past 30 days (item borrowed from GSHS). The response options were “Never”, “Rarely”, “Sometimes”, “Most of the time”, and “Always”.

Participants’ depressive symptoms over the past two weeks were assessed using the major depression inventory (MDI); which has been found reliable (Cronbach’s alpha = 0.83) and valid (Loevinger’s H coefficient for MDI’s 10 items was 0.38 confirming a unidimensional structure) to use among adolescents in this setting [51]. Both total raw scores from MDI and diagnostic classifications (i.e. 0–20 (No or doubtful depression, 21–25 (mild depression), 26–30 (moderate depression) and 31–50 (severe depression) [52] are utilized in our analysis.

### Statistical analysis

All analyses were conducted in the STATA15 software package (StataCorp LLC). Latent class analysis (LCA) [53] was done to identify clustering based on the 13 behavioral factors among the five behavioral categories, as described earlier. LCA is a more person-centred finite mixture modeling approach and its more recent extensions allow for manipulation of ordinal, nominal, continuous and count data [54]. LCA classifies individuals from a heterogeneous population into smaller more homogeneous groups called latent classes or groups. Membership to these non-directly observed (latent) groups is inferred from the participants’ varying patterns of responses in the data. Participants were placed in respective latent classes based on their posterior probabilities of belonging to a particular latent class. For each cluster, the proportion (%) of specific behavioral factors was computed and differences across clusters were tested using Chi-square tests [55]. Six models varying from one to six latent classes were generated so as to select the model with the best goodness of fit indices. The Akaike information criterion (AIC) [56] and Bayesian information criterion (BIC) [57] were utilized to identify the best models. Lower AIC and BIC values indicate better model fitting [58]. Entropy was also assessed to indicate the level of separation between classes. Higher values of normalized entropy represent a better fit whereby values greater than 0.80 indicate that the latent classes are highly discriminating [59, 60]. Meaningfulness and interpretability of the classes was also taken into account while determining the suitable number of classes. Descriptive analysis of means and percentages were used to summarize the socio-demographic characteristics of the adolescents in each cluster.

Stepwise ordinal logistic regression with nonparametric bootstrapping [61] at 95% confidence interval was utilized to identify factors associated with behavioral cluster (latent class) membership. The initial model was fitted with the variables of age (in years), sex, education level, current school attendance (yes/no), depressive symptoms over the past 2 weeks, residence (rural/urban/peri-urban) and level of parental monitoring in the past 30 days. The specified re-sampling clusters in this stepwise ordinal logistic regression model were the sub-locations used during stratified random sampling.

Overall, there was 9.9% of missing data for only 9 out of the 13 behavioral variables included in our analysis. Little’s MCAR test [62] indicated that these data were missing completely at random (P>0.05). Multiple imputation was used for missing data on these individual behavioral variables due to non-response [63].

## Results

The final sample comprised 1060 adolescents with a mean age of 15.7 years (SD = 1.9), though the majority (66.1%) were in the older adolescent age-group of 15–19 years. The proportion of males among older (15-19years) and younger (13-14years) adolescents was similar (i.e. 52.6% versus 53.5%). Most (79.2%) of the participants had a primary school level of education. Most adolescents (77.4%) resided in rural settings of Kilifi County.

Three behavioral clusters were optimal among the 6 different models on the basis of normalized entropy values (i.e. highest entropy value) and the BIC and AIC criteria (i.e. lowest BIC and AIC) (see Table 1).

**Table 1:** Model fit information for the latent class models from 1–6 clusters (n = 1060).

Number of clusters	Degrees of freedom	AIC	BIC	Entropy
**1**	21	15815.34	15919.62	
**2**	43	15233.03	15446.57	0.33
**3**	**65**	**15026.18**	**15418.97**	**0.70**
**4**	84	15070.41	15487.56	0.64
**5**	97	15028.11	15509.81	0.59
**6**	122	15035.81	15641.66	0.56

AIC: Akaike information criterion, BIC: Bayesian information criterion. The optimal latent class model is highlighted in bold

**Behavioral characteristics of the clusters.** Results on the behavioral characteristics of each cluster are presented in Table 2.

**Table 2:** A comparison of behavioral characteristics across the 3 behavioral clusters.

Variable	Total sample N (%) *n = 1060*	Cluster 1 (*Low risk-takers*) N (%) *n = 243 (22*.*9%)*	Cluster 2 (*Moderate risk-takers*) N (%) *n = 718 (67*.*8%)*	Cluster 3 (*High risk-takers*) N (%) *n = 99 (9*.*3%)*	P-Value
**Injury and violence related behavior**					
Being seriously injured during past 12 months (*Once or More than once)*	235 (22.2)	62 (25.5)^a^	129 (18.0)^c^	44 (44.4)^d^	<0.001
Was bullied during the past 30 days	278 (26.2)	49 (20.2)	186 (25.9)^c^	43 (43.4)^b^	<0.001
**Hygiene related behavior**					
Poor Oral hygiene (*cleaned teeth less than once a day or not at all or per day during the past 30 days*)	204 (19.3)	10 (4.1)^a^	168 (23.4)	26 (26.3)^b^	<0.001
Poor hand washing after toilet during past 30 days (rarely or never)	221 (20.8)	1 (0.4)^a^	184 (25.6)^e^	36 (36.4)^b^	<0.001
**Alcohol, tobacco and drug use behavior**					
Early alcohol drinking initiation *(13 years or less)*	116 (10.9)	0 (0.0)^a^	49 (6.8)^c^	67 (67.7)^b^	<0.001
Drank alcohol during past 30 days	59 (5.6)	0 (0.0)	0 (0.0)^c^	59 (59.6)^b^	<0.001
Early cigarette smoking initiation *(13 years or less)*	25 (2.4)	0 (0.0)	6 (0.8)^c^	19 (19.2)^b^	<0.001
Smoked cigarettes during past 30 days	47 (4.4)	1 (0.4)	0 (0.0)^c^	46 (46.5)^b^	<0.001
Lifetime marijuana	27 (2.6)	0 (0.0)	3 (0.4)^c^	24 (24.2)^b^	<0.001
**Physical activity**					
Physically active for at least 60 minutes during past week (*5–7 days*)	321 (30.3)	106 (43.6)^a^	181 (25.2)^f^	34 (34.3)	<0.001
5 or more sedentary hours on a typical day	105 (9.9)	51 (20.9)^a^	39 (5.4)^c^	15 (15.2)	<0.001
**Dietary behavior**					
Fruit consumption per day during past month (*3 times or more*)	268 (25.3)	207 (85.2)^a^	44 (6.1)^c^	17 (17.2)^b^	<0.001
Eating fast foods on 5–7 days during past week	211 (19.9)	95 (39.1)^a^	90 (12.5)	26 (26.3)^b^	<0.001

Chi Square test (for categorical variables);

^**a**^ p<0.001 (cluster 1 *vs*. 2);

^**b**^ p<0.001 (cluster 1 *vs*. 3);

^**c**^ p<0.001 (cluster 2 *vs*. 3);

^**d**^ p = 0.001 (cluster 1 *vs*. 3);

^**e**^ p = 0.002 (cluster 2 *vs*. 3);

^**f**^ p = 0.03 (cluster 2 *vs*. 3)

*Cluster 1 (low risk-takers)* was characterized by adolescents who reported abstinence from substance use (alcohol, tobacco, marijuana); much better oral hygiene and handwashing behavior as compared to other cluster members; and reported the highest daily fruit intake (85% reported eating fruits 3 times or more per day). Their reported involvement in injury and violence was also much lower than that reported by adolescents in cluster 3 and more adolescents in cluster 1 reported engaging in at least an hour of physical exercise on 5–7 days in the past week compared to those of cluster 2 (p = 0.03). However, the adolescents of cluster 1 (low-risk takers) consumed fast foods more frequently than adolescents from other clusters and spent more sedentary hours on a typical day than adolescents in cluster 2.

*Cluster 2 (moderate risk-takers)* comprised adolescents that reported very low substance use, had the least reports of serious injury (18%), least hours of sedentary activities on a typical day, and reported much lower consumption of fast foods than those of cluster 1 (p<0.001). Their reported oral hygiene and handwashing behavior was only better than that of adolescents in cluster 3. Adolescents in cluster 2, however, had the least daily fruit consumption (only 6% reported eating fruits 3 times or more) and the lowest proportion (25%) of adolescents who engaged in active physical activity for 5–7 days per week.

*Cluster 3 (high risk-takers)* was characterized by having the highest reports of substance use behavior (both early initiation and current use), poorest oral hygiene and handwashing practices, and its members reported the highest involvement in injury and violence (44%). Adolescents in cluster 3 also reported the second-lowest daily fruit consumption and spending more sedentary time than those in cluster 2.

### Socio-demographic characteristics of behavioral clusters

The socio-demographic characteristics of adolescents in each cluster are summarized in Table 3.

**Table 3:** Socio-demographic and psychosocial characteristics of the 3 behavioral clusters.

Variable	Total sample N (%) or mean (SD) *n = 1060*	Cluster 1 (*Low risk-takers*) N (%) or mean (SD) *n = 243*	Cluster 2(*Moderate risk-takers*) N (%) or mean (SD) *n = 718*	Cluster 3 (H*igh risk-takers*) N (%) or mean (SD) *n = 99*	P-Value
**Age** (years)	15.7 (SD. 1.9)	15.3 (SD 1.9)^**a**^	15.7 (SD 2.0)^**f**^	16.6 (1.7)^**i**^	<0.001
**Adolescent age group**		p^**b**^	p ^**f**^	p^**i**^	<0.001
Young adolescents (13-14y)	359 (33.9)	102 (42.0)	244 (34.0)	13 (13.1)	
Older adolescents (15-19y)	701 (66.1)	141 (58.0)	474 (66.0)	86 (86.9)	
**Sex**		p^**c**^	p^**f**^	p^**i**^	<0.001
Female	499 (47.1)	132 (54.3)	350 (48.7)	17 (17.2)	
Male	561 (52.9)	111 (45.7)	368 (51.3)	82 (82.8)	
**Residence**		p^**d**^	p^**g**^	p^**j**^	0.336
Rural	820 (77.4)	194 (79.8)	551 (76.7)	75 (75.8)	
Urban	140 (13.2)	24 (9.9)	104 (14.5)	12 (12.1)	
Peri-urban	100 (9.4)	25 (10.3)	63 (8.8)	12 (12.0)	
**Depressive symptoms**		p^**e**^	p^**h**^	p^**k**^	0.004
No depression	992 (93.6)	221 (90.9)	685 (95.4)	86 (86.9)	
Mild depression	37 (3.5)	12 (4.9)	20 (2.8)	5 (5.1)	
Moderate depression	15 (1.4)	6 (2.5)	6 (0.8)	3 (3.0)	
Severe depression	16 (1.5)	4 (1.7)	7 (1.0)	5 (5.0)	
**Currently attending school**		p^**m**^	p^**f**^	p^**i**^	<0.001
Yes	926 (87.5)	219 (90.1)	636 (89.1)	68 (69.4)	
No	132 (12.5)	24 (9.9)	78 (10.9)	30 (30.6)	
**Parental monitoring during past 30 days**		p^**n**^	p^**o**^	p^**i**^	<0.001
Always	179 (16.9)	62 (25.5)	106 (14.8)	11 (11.2)	
Most of the time	246 (23.2)	80 (32.9)	152 (21.1)	14 (14.3)	
Sometimes	147 (13.8)	23 (9.5)	106 (14.8)	18 (18.4)	
Rarely	197 (18.6)	32 (13.2)	140 (19.5)	25 (25.5)	
Never	288 (27.2)	46 (18.9)	212 (29.6)	30 (30.6)	

Bonferroni correction for post-hoc analysis in ANOVA (for continuous variables);

^**a**^ p = 0.003 (cluster 1 *vs*. 2);

^**f**^ p<0.001 (cluster 2 *vs*. 3);

^**i**^ p<0.001 (cluster 1 *vs*. 3). Chi Square test (for categorical variables);

p^**b**^ = 0.025 (cluster 1 *vs*. 2);

p^**c**^ = 0.133 (cluster 1 *vs*. 2);

p^**d**^ = 0.168 (cluster 1 *vs*. 2);

p^**m**^ = 0.648 (cluster 1 *vs*. 2);

p^**n**^ <0.001 (cluster 1 *vs*. 2);

p^**e**^ = 0.057;

p^**f**^ <0.001 (cluster 2 *vs*. 3);

p^**g**^ = 0.493 (cluster 2 *vs*. 3);

p^**h**^ = 0.001 (cluster 2 *vs*. 3);

p^**o**^ = 0.281 (cluster 2 *vs*. 3);

p^**i**^ <0.001 (cluster 1 *vs*. 3);

p^**j**^ = 0.703 (cluster 1 *vs*. 3);

p^**k**^ = 0.346 (cluster 1 *vs*. 3). SD: Standard deviation

The composition of these clusters ranged from 9% (high risk-takers) to 68% (moderate risk-takers) of the 1060 adolescents who participated in the study. There were significant age differences across the clusters with the mean age being highest (16.6 years) among adolescents in cluster 3 (high risk substance users) and lowest (15.3 years) for cluster 1 (low risk takers). The majority of the high risk-takers (cluster 3) were male adolescents (83%; (p<0.001)). In the other clusters, the composition by sex was almost balanced. The reported burden of mild to severe depressive symptoms among high risk-takers (8%) was 4 times higher than that in cluster 2 and twice that in cluster 1 (p = 0.004). In comparison to clusters 1 and 2, there was a greater proportion of adolescents (30.6%) in cluster 3 (High risk-takers) who were currently not attending school. Adolescents in the high risk-takers’ cluster also had the highest reports (56.1%) of sub-optimal parental monitoring during the past 30 days (i.e. rarely or never). Overall, 77.4% of the adolescents in each cluster were rural residents and there were no significant differences across the clusters.

### Factors associated with behavioral cluster membership

Table 4 summarizes the results of factors associated with cluster membership.

**Table 4:** Factors associated with membership to behavioral clusters from multivariate stepwise ordinal logistic regression (n = 1,058).

Factors	Odds Ratio	95% confidence interval	P-Value
Age (years)	1.15	1.08, 1.23	<0.001
**Sex**			
Female (Reference)			
Male	2.06	1.53, 2.75	<0.001
**Depressive symptoms**			
No depressive symptoms over the past 2 weeks (Reference)			
Depressive symptoms over the past 2 weeks	1.04	1.01, 1.06	0.005
Parental monitoring during past 30 days			<0.001
Never (Reference)			
Most of the time	0.45	0.34, 0.60	<0.001
Always	0.44	0.33, 0.58	<0.001
**Currently attending school**			
Yes (Reference)			
No	2.13	1.37, 3.31	0.001

Ordinal outcome with increasing levels of risk taking (*Low risk takers*, *Moderate risk takers*, *High risk takers*).

Data from two participants was not available due to list-wise deletion.

Age was associated with cluster membership with the odds of belonging to a higher risk-taking cluster increasing with the age of the adolescent. Sex was also strongly associated with cluster membership (p<0.001). The odds of belonging to one of the higher risk clusters (i.e. moderate or high risk-takers) relative to the cluster 1 increased with higher scores on the MDI (a measure of depressive symptoms) (p<0.001). Parental monitoring was also associated with cluster membership in that the odds of belonging to one of the higher risk clusters (i.e. moderate or high risk-takers) relative to the cluster 1 were lower for adolescents whose parent/guardian always or most of the time knew what the adolescents did in their free time compared to those whose parent/guardian never knew what they did during their free time. The odds of belonging to a higher risk-taking cluster were higher among out of school adolescents (p = 0.001).

## Discussion

This study demonstrates that there is clustering of injury, substance use, poor hygiene, and suboptimal physical activity and dietary behavior among adolescents living in Kilifi, Kenya. Three distinct latent HRB clusters were identified. Three studies on HRB clustering conducted among adolescents from the Netherlands [64], China [65] and the USA [66] generated four behavioral clusters. The difference in the number of clusters generated may be due to the variation in the forms of behavioral factors upon which clustering was conducted and due to disparity in contextual factors. The variation in magnitude of risk-taking across the clusters in our study is consistent with results from the Dutch study which showed that one of their 4 clusters comprised higher risk behavior coupled to negative health outcomes [64]. In our study, the 2 healthier clusters (clusters 1 and 2) had some occurrence of injury and physical inactivity but the distinctive characteristic of the moderate risk cluster (cluster 2) was their poor hygiene. We propose that the differences in hygiene may indicate that adolescents in the moderate risk cluster were more disadvantaged, but unfortunately data to elucidate this were not collected.

Another key finding was that risky behaviors associated with communicable and non-communicable diseases clustered together. The clustering of poor hygiene, smoking, and inadequate fruit/vegetable consumption was also seen among Chinese adolescents [67] and clustering of substance use and sexual risk behavior among adolescents in the USA [66]. The implications of these findings are that behavioral interventions should simultaneously address communicable and NCDs-related risk behaviors of adolescents. Family-based multiple component interventions targeting adolescents and their parents have been shown to address communicable and NCDs-related HRB simultaneously [11]. Such interventions for instance comprise components which reinforce adolescents’ refusal and problem solving skills, increase parental awareness of the risks faced by their adolescents and help to improve adolescent-parent communication [11]. However, more research is needed to more fully understand the specific mechanisms that underlie the clustering of communicable and NCD-related risk behaviors as this could support the design of the most effective interventions.

We found a high burden of injury and violence, which overlapped with high levels of substance use, predominantly among adolescents in Cluster 3 (high risk-takers). A strong association between exposure to bullying and substance use (alcohol, tobacco, and drugs) among adolescents has been reported from a multi-site study among 8 African countries, Kenya inclusive [24]. Substance use, especially alcohol use, has been described as an important cross-cutting predisposing factor to unintentional injury and violence during adolescence [68]. With the trends towards increasing and earlier age of initiation of substance use among Kenyan youths [37], there is need for more comprehensive substance use prevention and harm reduction interventions that concurrently address injury and violence.

Another key finding is that age and sex are important socio-demographic characteristics associated with the co-occurrence of HRB. Similar to our findings, older age and being male were associated with membership of riskier behavioral clusters among Chinese adolescents [67] and adolescents from Bahamas [69], though these factors were not associated with cluster membership among Dutch adolescents [64]. Our findings on the association of age and sex with co-occurrence of HRB highlight the importance of ensuring that interventions designed to address multiple HRB during adolescence are both developmentally appropriate and gender sensitive.

Severity of depressive symptoms among adolescents from Kilifi was associated with higher risk clusters. This relationship may be bi-directional, however, investigating the directionality was beyond the scope of this cross-sectional study. This finding is consistent with results from other adolescent studies that have shown a link between depressive symptoms and engagement in multiple forms of HRB such as substance use, risky sex and sedentary behavior during adolescence [70–72]. A plausible mechanism linking depressive symptoms to HRB clustering is experience of early childhood adverse life events. Indeed, Kenyan studies have found that adolescents who experienced adverse events such as extreme household poverty, living with caregivers who abuse substances, living with sick caregivers and experience of family conflict were more likely to engage in various forms of delinquent and health risk behavior [45, 73, 74], as well as experience psychological problems like anxiety and depression [74, 75]. These findings highlight the need for inclusion of the prevention and management of common mental health disorders in interventions addressing HRB. Moreover, it provides the impetus for longitudinal work to investigate causal pathways and identify the most strategic timing and aspects of intervention.

We found that parental monitoring of adolescents was associated with a lower risk of being a member of a higher risk cluster, though this relationship may also be in either direction. This finding underscores an important and urgent need for rigorous research to examine parenting behavior and its underlying factors so as to identify specific barriers and opportunities for promoting supportive parenting behavior among adolescents’ caregivers in Kilifi. Other studies report that parent/caregiver knowledge of their children’s whereabouts, activities, and associates (parental monitoring) builds resilience, moderates peer-influenced risk behavior and its effects persist into late adolescence [76]. This finding is suggestive that parental monitoring interventions may address HRB among adolescents in Kilifi. Some potential components of such interventions include fostering parent-child communication, improving parental engagement in adolescents’ activities like school activities, and teaching adolescents interpersonal skills [77, 78]. However, these intervention components require testing and tailoring to the context of Kilifi.

Finally, we found that school attendance played a protective role against membership to the higher risk clusters. Indeed, the school environment is a key setting for promoting child and adolescent health and safety as has been reflected in Kenya’s national school health policy and strategic implementation plan [79, 80]. Nutrition health, sanitation and hygiene, disease prevention, child rights, gender issues, and life skills are among some key thematic areas prioritized for strategic implementation in Kenyan school environment [80]. Thus, adolescents who attend school are more likely to benefit from these various health promotion services and structures which potentially offers significant protective benefits against multiple forms of HRB. Conversely, our finding also emphasizes an important need to improve efforts targeting adolescents who do not attend school, as these may face considerable risk for multiple forms of HRB. There is need for multi-sectoral coordination and poverty-intervention programs that seek to change individuals, families, school systems and public policies so as to address poor educational outcomes [79, 81]. Also from a broader perspective, an urgent need to address underlying determinants of adolescent HRB, such as household poverty, complacency in law enforcement, and potentially harmful social norms and customs like early marriages and patriarchy, has been emphasized among other studies conducted in Kilifi [45, 82]. Flexible adolescent-friendly service delivery models that reach beyond the school and health facility environment are required in this setting.

### Strengths and limations

The main strength of this study is the breadth of risky behaviors that were considered and the use of a robust analytical approach (latent class analysis) to understand HRB profiles based on a variety of behavioral outcomes simultaneously. To the best of our knowledge, there has not been any study applying this approach in understanding adolescent HRB within Kenya and very few elsewhere in SSA. Although permission was specifically requested to explore sexual behavior of the study participants, ethical approval was granted to collect data on sexual behavior only among the older youths aged 18 years and above. Thus, sexual risk behavior was not included as it was only assessed in older youths who are not part of our adolescent sample. Nonetheless, our study included both communicable and NCD-related behaviors which reflects the double burden of morbidity within SSA [83]. The study had an acceptable (70%) participant response.

However, HRB was self-reported and this may have introduced some level of social-desirability bias, which may have been different according to age, sex or other risk factors. Nonetheless, the use of ACASI likely reduced reporting bias [84]. There were a few participants (less than 3%) that needed some minimal help, especially with maneuvering from one item to the next in some of the sections of the ACASI. Its plausible that during assistance by the research assistant, some level of self-desirability bias may have been introduced. Our categorization of participants’ residence into rural, peri-urban and urban setting based on the KHDSS sub-locations may reflect the general socio-economic status of the sub-locations. However, our study lacked household-level measures of socio-economic status such as household income, parental level of education or employment, which may have an important role in HRB clustering. Its also probable that re-categorization of HRBs may have introduced some level of assessment bias especially through assortment of precise details within the data. Also, this being a cross-sectional study, it was not possible to establish the direction of the associations that were found. Besides, a more in-depth understanding of the mechanisms underlying clustering of HRB in this sub-population would require a more explorative research approach to complement the quantitative findings from this study.

We used the AIC and BIC criteria to identify the optimal latent classes. However, these criteria have some limitations, especially the AIC [85]. Nonetheless, the values of the BIC (which is considered superior to AIC) in our analysis indicated that our model choice (3 latent classes) was most optimal. Also, latent class analysis is a person-centered approach and as such, our results may not be fully generalizable to other samples of adolescents.

## Conclusions

There is constellation of HRB related to communicable and non-communicable diseases among adolescents in rural coastal Kenya and thus multi-component health behavior interventions which simultaneously address all HRB may contribute to greater public health benefit. These interventions are also likely to be more effective when explanatory factors and mechanisms underlying HRB clustering are identified so that adolescents’ specific needs are addressed and beneficial intervention components are tailored to the local context. To tailor interventions to this adolescent population rapid situation analyses on adolescents’ and parenting needs within the study setting are needed in order to identify and incorporate relevant intervention components. Some promising interventions components include building parenting skills and communication within the family, strengthening adolescents’ problem-solving skills, strengthening resilience in the face of adversity, strengthening family connectedness and providing pyscho-education which can target contextually relevant sources of mental illness. There is also need for improved targeting of adolescents who drop out of school as well as those at risk of school dropout [86, 87]. This is because majority of the adolescent health research and interventions have habitually targeted school-attending adolescents [11]. Besides, the urgent need for increased prioritization of research and investment in programmes focusing on Kenya’s most-at-risk adolescents is well documented [88]. It is plausible that the adolescents who were vulnerable to multiple forms of HRB within our study setting comprised most-at-risk adolescents, whose specific needs necessitate further investigation so as to tailor responsive interventions and services. Adolescent health services or interventions targeting HRB need to stretch beyond focusing on HRB by also including mental health management. Future studies need to examine mechanisms that underlie the clustering of communicable and NCD-related HRB during adolescence.

## Supporting information

S1 File (DOCX)

S1 Questionnaire Young people health survey questionnare (English studio version). (DOCX)

S2 Questionnaire Young people health survey questionnare (Giriyama studio version). (DOCX)

S3 Questionnaire Young people health survey questionnare (Swahili studio version). (DOC)
