# Supplementary material for: Clustering of health risk behaviors among adolescents in Kilifi, Kenya, a rural Sub-Saharan African setting
Source: PLoS One. 2020 Nov 12;15(11):e0242186. doi: 10.1371/journal.pone.0242186 (PMC7660520; doi:10.1371/journal.pone.0242186)
Supplement: S1 File — (DOCX) [file pone.0242186.s001.docx]

**Supplementary materials:** Items/questions on behavioral factors which were utilized in the analysis of health risk behavior clustering

**Injury or violence-related behavior:**

*The next 3 questions ask about serious injuries that happened to you. An injury is serious when it makes you miss at least one full day of usual activities (such as school, sports, or a job) or requires treatment by a doctor or nurse.*

During the past 12 months, how many times were you seriously injured?

1. 0 times
2. 1 time
3. 2 or 3 times
4. 4 or 5 times
5. 6 or 7 times
6. 8 or 9 times
7. 10 or 11 times
8. 12 or more times

*The next 2 questions ask about bullying. Bullying occurs when a student or group of students say or do bad and unpleasant things to another student. It is also bullying when a student is teased a lot in an unpleasant way or when a student is left out of things on purpose. It is not bullying when two students of about the same strength or power argue or fight or when teasing is done in a friendly and fun way.*

During the past 30 days, on how many days were you bullied?

1. 0 days
2. 1 or 2 days
3. 3 to 5 days
4. 6 to 9 days
5. 10 to 19 days
6. 20 to 29 days
7. All 30 days

**Substance use:**

*The next 6 questions ask about cigarette and other tobacco use*

How old were you when you first tried smoking a cigarette?

1. I have never smoked cigarettes
2. 7 years old or younger
3. 8 or 9 years old
4. 10 or 11 years old
5. 12 or 13 years old
6. 14 or 15 years old
7. 16 or 17 years old
8. 18 years or older

During the past 30 days, on how many days have you smoked cigarettes?

1. 0 days
2. 1 or 2 days
3. 3 to 5 days
4. 6 to 9 days
5. 10 to 19 days
6. 20 to 29 days
7. All 30 days

*The next 4 questions ask about drinking alcohol. Alcohol includes drinks such as mnazi, changaa, beer, vikali, and pombe. Drinking alcohol does not include drinking a few sips of wine for religious purposes. A “drink” is a glass of wine, a bottle of beer, a small glass of liquor, or a mixed drink.*

How old were you when you had your first drink of alcohol other than a few sips?

1. Never had a drink of alcohol other than a few sips
2. Never taken alcohol at all
3. 7 years old or younger
4. 8 or 9 years old
5. 10 or 11 years old
6. 12 or 13 years old
7. 14 or 15 years old
8. 16 or 17 years old
9. 18 years or older

During the past 30 days, how many days did you have at least one rink containing alcohol?

1. 0 days
2. 1 or 2 days
3. 3 to 5 days
4. 6 to 9 days
5. 10 to 19 days
6. 20 to 29 days
7. All the 30 days

*The next 3 questions ask about drug use. This includes using marijuana, amphetamines, cocaine, inhalants.*

During your life, how many times have you used marijuana (also called bhang, bosa, bomu, dom, holy, herb, hashish)

1. 0 times
2. 1 or 2 times
3. 3 to 9 times
4. 10 to 19 times
5. 20 or more times

**Hygiene behavior**:

*The next 4 questions ask about cleaning your teeth and washing your hands*

During the past 30 days, how many times per day did you usually clean or brush your teeth?

1. Did not clean or brush your teeth during the past 30 days
2. Less than 1 time per day
3. Once a day
4. Twice a day
5. Thrice a day
6. 4 or more times per day

During the past 30 days, how often did you wash your hands after using the toilet or latrine?

1. Never
2. Rarely
3. Sometimes
4. Most of the time
5. Always

**Physical activity:**

*The next 3 questions ask about physical activity. Physical activity is any activity that increases your heart rate and makes you breathe hard. Physical activity can be done in sports, playing with friends, or walking to school. Some examples of physical activity are running, fast walking, biking, dancing, football, swimming, and handball*.

During the past 7 days, on how many days were you physically active for a total of 60 minutes per day? (Add up all the time you spent in any kind of physical activity each day).

1. 0 days
2. 1 day
3. 2 days
4. 3 days
5. 4 days
6. 5 days
7. 6 days
8. 7 days

*The next question asks about the time that you spend mostly sitting when you are not in school or doing homework.*

How much time do you spend during a typical or usual day sitting and watching television, talking with friends, or doing other sitting activities such as storytelling?

1. Less than 1 hour per day
2. 1 to 2 hours per day
3. 3 to 4 hours per day
4. 5 to 6 hours per day
5. 7 to 8 hours per day
6. More than 8 hours per day

**Dietary behavior:**

During the past 30 days, how many times per day did you usually eat fruits such as oranges, pawpaw, pineapple, mangoes, coconuts, kunazi, guavas, lemons, fruit salad?

1. Did not eat fruits during the past 30 days
2. Less than one time per day
3. 1 time per day
4. 2 times per day
5. 3 times per day
6. 4 times per day
7. 5 or more times per day

During the past 7 days, on how many days did you eat food from a fast-food restaurant, such as chips, viazi karai, mahamuri, chapatis?

1. 0 days
2. 1 day
3. 2 days
4. 3 days
5. 4 days
6. 5 days
7. 6 days
8. 7 days
