# Supplementary material for: Clustering of health risk behaviors among adolescents in Kilifi, Kenya, a rural Sub-Saharan African setting
Source: PLoS One. 2020 Nov 12;15(11):e0242186. doi: 10.1371/journal.pone.0242186 (PMC7660520; doi:10.1371/journal.pone.0242186)
Supplement: S1 Questionnaire — (DOCX) [file pone.0242186.s002.docx]

**YOUNG PEOPLE HEALTH SURVEY QUESTIONNARE (ENGLISH STUDIO VERSION)**

| **Anthropometric measurements**  **(Please remove your shoes and extra clothes eg, jackets, coat, sweater, pullover so that I can measure your weight and height** | | |
| --- | --- | --- |
| Height | [_][_][_].[_] cm | |
| MUAC | [_][_] . [_]cm | |
| Weight | [_][_] . [_]kg | |
| 1. Are you in school? | If Yes select A  If No select B | |
| 1. What grade/class/ standard/form are you? | - If you are in Class 3; select A - If you are in Class 4; select B - If you are in Class 5; select C - If you are in Class 6; select D - If you are in Class 7; select E - If you are in Class 8; select F - If you are in form 1; select G - If you are in Form 2; select H - If you are in Form 3; select I - If you are in Form 4; select J - If you are in Tertiary; select K - If Other; Select L and specify which | |
| 1. The last time you were unwell, where did you seek help? | - If you never sought help; select A - If you went to a Government facility; select B - If you went to a Private facility; select C - If you went to a Drug shops; select D - If you went to a Chemist; select E - If you went to a Traditional healer; select F - If Other select; G | |
| **The next 4 questions ask about cleaning your teeth and washing your hands.** | | |
| 1. During the past 30 days, how many times per day did you usually clean or brush your teeth? | - If you did not clean or brush your teeth during the past 30 days; select A - If Less than 1 time per day; select B - If once a day; select C - If twice a day; select D - If thrice a day; select E - If 4 or more times per day; select F | |
| 1. During the past 30 days, how often did you wash your hands before eating? | - If Never; select A - If Rarely; select B - If Sometimes; C - If Most of the time; D - If Always; select E | |
| 1. During the past 30 days, how often did you wash your hands after using the toilet or latrine? | - If Never; select A - If Rarely; select B - If Sometimes; C - If Most of the time; D - If Always; select E | |
| 1. During the past 30 days, how often did you use soap when washing your hands? | - If Never; select A - If Rarely; select B - If Sometimes; C - If Most of the time; D - If Always; select E | |
| **The next 5 questions ask about what you might eat and drink.** | | |
| 1. During the past 30 days, how often did you go hungry because there was not enough food in your home? | - If Never; select A - If Rarely; select B - If Sometimes; select C - If Most of the time; select D - If Always; select E |  |
| 1. During the past 30 days, how many times per day did you usually eat fruit, such as oranges, pawpaw, pineapple, mangoes, coconuts, kunazi, guavas, lemons, fruit salad | - If you did not eat fruit during the past 30 days; select A - If Less than one time per day; select B - If 1 time per day; select C - If 2 times per day; select D - If 3 times per day; select E - If 4 times per day; select F - If 5 or more times per day; select G |  |
| 1. During the past 30 days, how many times per day did you usually eat vegetables, such as cabbages, sukuma wiki, carrots, mchicha, mnafu, saga, mrenda, | - If you did not eat vegetables during the past 30 days; select A - If Less than one time per day; select B - If 1 time per day; select C - If 2 times per day; select D - If 3 times per day; select E - If 4 times per day; select F - If 5 or more times per day; select G |  |
| 1. During the past 30 days, how many times per day did you usually drink carbonated soft drinks, such as Sodas,   (Do not include diet soft drinks.) | - If you did not drink carbonated soft drinks during the past 30 days; select A - If Less than one time per day; select B - If 1 time per day; select C - If 2 times per day; select D - If 3 times per day; select E - If 4 times per day; select F - If 5 or more times per day; select G |  |
| 1. During the past 7 days, on how many days did you eat food from a fast food restaurant, such as chips, viazi karai, mahamuri, chapatis ? | - - - For 0 days; select A     - For 1 day; select B     - For 2 day; select C     - For 3 days; select D     - For 4 days; select E     - For 5 days; select F     - For 6 days; select G     - For 7 days; select H |  |
| **The next 4 questions ask about drinking alcohol. This includes drinking mnazi, changaa, beer, pombe. Drinking alcohol does not include drinking a few sips of wine for religious purposes. A “drink” is a glass of wine, a bottle of beer, a small glass of liquor, or a mixed drink.** | | |
| 1. How old were you when you had your first drink of alcohol other than a few sips? | - If you have never had a drink of alcohol other than a few sips; select A - If you have never taken alcohol at all; select B - If when you were 7 years old or younger; select C - If when you were 8 or 9 years old; select D - If when you were 10 or 11 years old; select E - If when you were 12 or 13 years old; select F - If when you were 14 or 15 years old; select G - If when you were 16 or 17 years old; select H - If when you were 18 years old or older; select I | |
| 1. During the past 30 days, how many days did you have at least one drink containing alcohol? | 1. If 0 days select A 2. If 1 or 2 days select B 3. If 3 to 5 days select C 4. If 6 to 9 days select D 5. If 10 to 19 days select E 6. If 20 to 29 days: select F 7. If All 30 days; select G | |
| 1. During the past 30 days, on the days you drank alcohol, how many drinks did you usually drink per day? | - If you did not drink alcohol during the past 30 days select A - If Less than one drink; select B - If 1 drink; select C - If 2 drinks; select D - If 3 drinks; select E - If 4 drinks; select F - If 5 or more drinks; select G | |
| 1. During the past 30 days, how did you usually get the alcohol you drank? | - If you did not drink alcohol during the past 30 days; select A - If you bought it in a store, shop, or from a street vendor; select B - If you gave someone else money to buy it for you; select C - If you got it from your friends; select D - If you got it from your family; select E - If you stole it or got it without permission; select F - If you got it some other way; select G | |
| **Staggering when walking, not being able to speak right, and throwing up are some signs of being really drunk.** | | |
| 1. During your life, how many times did you drink so much alcohol that you were really drunk? | - If 0 times; select A - If 1 or 2 times; select B - If 3 to 9 times; select C - If 10 or more times; select D | |
| 1. During your life, how many times have you got into trouble with your family or friends, missed school, or got into fights, as a result of drinking alcohol? | - If 0 times; select A - If 1 or 2 times; select B - If 3 to 9 times; select C - If 10 or more times; select D | |
| **The next 3 questions ask about drug use. This includes using marijuana, amphetamines, cocaine, inhalants** | | |
| 1. How old were you when you first used drugs? | - If you have never used drugs; select A - If you were 7 years old or younger; select B - If you were 8 or 9 years old; select C - If you were 10 or 11 years old; select D - If you were 12 or 13 years old; select E - If you were 14 or 15 years old; select F - If you were 16 or 17 years old; select G - If you were 18 years old or older; select H |  |
| 1. During your life, how many times have you used marijuana (also called bhang, bosa, bomu, dom”, holy, herb, hashish) | - If 0 times; select A - If 1 or 2 times; select B - If 3 to 9 times; select C - If 10 to 19 times; select D - If 20 or more times; select E |  |
| 1. During your life, how many times have you used sniffing glue | - If 0 times; select A - If 1 or 2 times; select B - If 3 to 9 times; select C - If 10 to 19 times; select D - If 20 or more times; select E |  |
|  | | |
| **The following questions ask about how you have been feeling over the past two weeks. Please put a tick in the box which is closest to how you have been feeling.**  **Major Depression Inventory (MDI)** | | |
| 1. Have you felt low in spirits or sad? | - If at no time select 0 - If Some of the time; select 1 - If Slightly less than half the time; select 3 - If Slightly more than half the time; select 3 - If Most of the time; select 4 - If all the time; select 5 | |
| 1. Have you lost interest in your daily activities? | - If at no time select 0 - If Some of the time; select 1 - If Slightly less than half the time; select 3 - If Slightly more than half the time; select 3 - If Most of the time; select 4 - If all the time; select 5 | |
| 1. Have you felt lacking in energy and strength? | - If at no time select 0 - If Some of the time; select 1 - If Slightly less than half the time; select 3 - If Slightly more than half the time; select 3 - If Most of the time; select 4 - If all the time; select 5 | |
| 1. Have you felt less self-confident? | - If at no time select 0 - If Some of the time; select 1 - If Slightly less than half the time; select 3 - If Slightly more than half the time; select 3 - If Most of the time; select 4 - If all the time; select 5 | |
| 1. Have you had a bad conscience or feelings of guilt? | - If at no time select 0 - If Some of the time; select 1 - If Slightly less than half the time; select 3 - If Slightly more than half the time; select 3 - If Most of the time; select 4 - If all the time; select 5 | |
| 1. Have you felt that life wasn’t worth living? | - If at no time select 0 - If Some of the time; select 1 - If Slightly less than half the time; select 3 - If Slightly more than half the time; select 3 - If Most of the time; select 4 - If all the time; select 5 | |
| 1. Have you had difficulty in concentrating, e.g. when reading the newspaper or watching television? | - If at no time select 0 - If Some of the time; select 1 - If Slightly less than half the time; select 3 - If Slightly more than half the time; select 3 - If Most of the time; select 4 - If all the time; select 5 | |
| 1. Have you felt very restless? | - If at no time select 0 - If Some of the time; select 1 - If Slightly less than half the time; select 3 - If Slightly more than half the time; select 3 - If Most of the time; select 4 - If all the time; select 5 | |
| 1. Have you felt subdued or slowed down? | - If at no time select 0 - If Some of the time; select 1 - If Slightly less than half the time; select 3 - If Slightly more than half the time; select 3 - If Most of the time; select 4 - If all the time; select 5 | |
| 1. Have you had trouble sleeping at night? | - If at no time select 0 - If Some of the time; select 1 - If Slightly less than half the time; select 3 - If Slightly more than half the time; select 3 - If Most of the time; select 4 - If all the time; select 5 | |
| 1. Have you suffered from reduced appetite? | - If at no time select 0 - If Some of the time; select 1 - If Slightly less than half the time; select 3 - If Slightly more than half the time; select 3 - If Most of the time; select 4 - If all the time; select 5 | |
| 1. Have you suffered from increased appetite? | - If at no time select 0 - If Some of the time; select 1 - If Slightly less than half the time; select 3 - If Slightly more than half the time; select 3 - If Most of the time; select 4 - If all the time; select 5 | |
| **The next 3 questions ask about physical activity. Physical activity is any activity that increases your heart rate and makes you breathe hard. Physical activity can be done in sports, playing with friends, or walking to school. Some examples of physical activity are running, fast walking, biking, dancing, football, swimming, handball,** | | |
| 1. During the past 7 days, on how many days were you physically active for a total of at least 60 minutes per day?   ADD UP ALL THE TIME YOU SPENT IN ANY KIND OF PHYSICAL ACTIVITY EACH DAY. | - For 0 days; select A - For 1 day; select B - For 2 days; select C - For 3 days; select D - For 4 days; select E - For 5 days; select F - For 6 days; select G - For 7 days select H | |
| 1. During the past 7 days, on how many days did you walk or ride a bicycle to or from school? | - For 0 days; select A - For 1 day; select B - For 2 days; select C - For 3 days; select D - For 4 days; select E - For 5 days; select F - For 6 days; select G - For 7 days select H | |
| 1. During this school year, on how many days did you go to physical education (PE) class each week? | - For 0 days; select A - For 1 day; select B - For 2 days; select C - For 3 days; select D - For 4 days; select E - For 5 or more days; select F | |
| **The next question asks about the time you spend mostly sitting when you are not in school or doing homework.** | | |
| 1. How much time do you spend during a typical or usual day sitting and watching television, talking with friends, or doing other sitting activities, such as storytelling, | - If Less than 1 hour per day; select A - If 1 to 2 hours per day; select B - If 3 to 4 hours per day; select C - If 5 to 6 hours per day; select D - If 7 to 8 hours per day; select E - If More than 8 hours per day; select F | |
| **The next 6 questions ask about your experiences at school and at home.** | | |
| 1. During the past 30 days, on how many days did you miss classes or school without permission? | - For 0 days; select A - For 1 or 2 days; B - For 3 to 5 days; select C - For 6 to 9 days; select D - For 10 or more days; select E | |
| 1. During the past 30 days, how often were most of the students in your school kind and helpful? | - If Never; select A - If Rarely; select B - If Sometimes; C - If Most of the time; D - If Always; select E | |
| 1. During the past 30 days, how often did your parents or guardians check to see if your homework was done? | - If Never; select A - If Rarely; select B - If Sometimes; C - If Most of the time; D - If Always; select E | |
| 1. During the past 30 days, how often did your parents or guardians understand your problems and worries? | - If Never; select A - If Rarely; select B - If Sometimes; C - If Most of the time; D - If Always; select E | |
| 1. During the past 30 days, how often did your parents or guardians really know what you were doing with your free time? | - If Never; select A - If Rarely; select B - If Sometimes; C - If Most of the time; D - If Always; select E | |
| 1. During the past 30 days, how often did your parents or guardians go through your things without your approval? | - If Never; select A - If Rarely; select B - If Sometimes; C - If Most of the time; D - If Always; select E | |
| **The next 6 questions ask about cigarette and other tobacco use** | | |
| 1. How old were you when you first tried smoking a cigarette? | - If you have never smoked cigarettes; select A - If 7 years old or younger; select B - If 8 or 9 years old; select C - If 10 or 11 years old; select D - If 12 or 13 years old; select E - If 14 or 15 years old; select F - If 16 or 17 years old; select G - If 18 years old or older; select H | |
| 1. During the past 30 days, on how many days have you smoked cigarettes? | - If 0 days; select A - If 1 or 2 days; select B - If 3 to 5 days; select C - If 6 to 9 days; select D - If 10 to 19 days; select - If 20 to 29 days; select F - If All 30 days; select G | |
| 1. During the past 30 days, on how many days have you used any tobacco products other than cigarettes, such as kiko, tobacco? | - If 0 days; select A - If 1 or 2 days; select B - If 3 to 5 days; select C - If 6 to 9 days; select D - If 10 to 19 days; select - If 20 to 29 days; select F - If All 30 days; select G | |
| 1. During the past 12 months, have you ever tried to stop smoking cigarettes? | - If you have never smoked cigarettes; select A - If you did not smoke cigarettes during the past 12 months; select B - If Yes; select C - If No; select D | |
| 1. During the past 7 days, on how many days have people smoked in your presence? | - For 0 days select A - If 1 or 2 days; select - If 3 or 4 days; select C - If 5 or 6 days; select D - If for All 7 days; select E | |
| 1. Which of your parents or guardians use any form of tobacco? | - If Neither; select A - If your father or male guardian; select B - If your mother or female guardian; select C - If Both; select D - If you do not know; select E | |
| **The next question asks about physical attacks. A physical attack occurs when one or more people hit or strike someone, or when one or more people hurt another person with a weapon (such as a stick, knife, or gun). It is not a physical attack when two students of about the same strength or power or age choose to fight each other for fun.** | | |
| 1. During the past 12 months, how many times were you physically attacked? | - If 0 times; select A - If 1 time; select B - If 2 or 3 times; select B - If 4 or 5 times; select C - If 6 or 7 times; select D - If 8 or 9 times; select E - If 10 or 11 times; select F - If 12 or more times; select G | |
| **The next 3 questions ask about serious injuries that happened to you. An injury is serious when it makes you miss at least one full day of usual activities (such as school, sports, or a job) or requires treatment by a doctor or nurse**. | | |
| 1. During the past 12 months, how many times were you seriously injured? | - If 0 times; select A - If 1 time; select B - If 2 or 3 times; select C - If 4 or 5 times; select D - If 6 or 7 times; select E - If 8 or 9 times; select F - If 10 or 11 times; select G - If 12 or more times; select H | |
| 1. During the past 12 months, what was the most serious injury that happened to you? | - If you were not seriously injured during the past 12 months; select A - If you had a broken bone or a dislocated joint; select B - If you had a cut or stab wound; select C - If had a concussion or other head or neck injury, was knocked out, or could not breathe; select D - If you had a gunshot wound; select E - If you had a bad burn; select F - If you were poisoned or took too much of a drug; select G - If Something else happened to you; select H | |
| 1. During the past 12 months, what was the cause of the most serious injury that happened to you? | - If you were not seriously injured during the past 12 months; select A - If you were in a motor vehicle accident or hit by a motor vehicle; select B - If you fell; select C - If Something fell on you or hit you; select D - If you were attacked or abused or was fighting with someone; select E - If you were in a fire or too near a flame or something hot; select F - If you inhaled or swallowed something bad select G - If something else caused your injury; select H | |
| **The next 2 questions ask about bullying. Bullying occurs when a student or group of students say or do bad and unpleasant things to another student. It is also bullying when a student is teased a lot in an unpleasant way or when a student is left out of things on purpose. It is not bullying when two students of about the same strength or power argue**  **or fight or when teasing is done in a friendly and fun way.** | | |
| 1. During the past 30 days, on how many days were you bullied? | - If 0 days; select A - If 1 or 2 days; select B - If 3 to 5 days; select C - If 6 to 9 days; select D - If 10 to 19 days; select - If 20 to 29 days; select F - If All 30 days; select G | |
| 1. During the past 30 days, how were you bullied most often? | - If you were not bullied during the past 30 days; select A - If you were hit, kicked, pushed, shoved around, or locked indoors; select B - If you were made fun of because of your race, nationality, or color; select C - If you were made fun of because of your religion; select D - If you were made fun of with sexual jokes, comments, or gestures; select E - If you were left out of activities on purpose or completely ignored - If you were made fun of because of how your body or face looks; select G - If you were bullied in some other way; select H | |
| **Now I’d like to ask about your marital status.** | | |
| 1. What is your current marital status? | - If Married; select A - If not married but living together with a partner of the opposite sex; select B - If widowed; select C - If divorced; select D - If separated because you and your spouse are not getting along; select E - If you have Never been married; select F | |
| 1. What was your age at first marriage? | - If less than or 13 years; select A - If 14 years; select B - If 15 years; select C - If 16 years; select D - If 17 years; select E - If 18 years or more; select F - If you don’t know; select G | |
| **Next, I want to talk to you about pregnancy** | | |
| 1. How many times have you been pregnant or gotten someone pregnant? | - If 0 times; select A - If once; select B - If 2 or more times; select C - If you do not know; select D | |
| 1. How old were you when you first got pregnant? (Females) | - If less than or 13 years; select A - If 14 years; select B - If 15 years; select C - If 16 years; select D - If 17 years; select E - If 18 years or more; select F - If you don’t know; select G | |
| 1. What happened to the pregnancy? | - If delivered live baby; select A - If still pregnant; select B - If it was a still birth; select C - If it resulted in an abortion or miscarriage; select D - If you do not know; select E | |

| **This last section is about sexual behaviours***.* | |
| --- | --- |
| 1. Have you ever had sex? | - If yes; select A - If no; select B |
| 1. How old were you when you had sex for the first time? | - If you have never had sexual intercourse; select A - If you were 11 years old or younger; select B - If you were 12 years old; select C - If you were 13 years old; select D - If you were 14 years old; select E - If you were 15 years old select F - If you were 16 or 17 years old; select G - If you were 18 years old or older; select H |
| 1. During your life, with how many people have you had sex? | - If you have never had sexual intercourse; select A - If 1 person; select B - If 2 people; select C - If 3 people; select D - If 4 people; select E - If 5 people; select F - If 6 or more people; select G |
| 1. The last time you had sex; did you or your partner use a condom or [mpira, CD]? | - If you have never had sexual intercourse; select A - If yes; select B - If no; select C |
| 1. The last time you had sex, did you or your partner use any other method of birth control, such as withdrawal, rhythm (safe time), birth control pills, or any other method to prevent pregnancy? | - If you have never had sexual intercourse; select A - If yes; select B - If no; select C - If you do not know; select D |
| 1. Have you ever been tricked into having sex when you did not want to? | - If Yes; select A - If No; select B |
| 1. Have you ever been locked in a room to have sex when you did not want to? | - If Yes; select A - If No; select B |
| 1. Have you ever been physically forced to have sex when you did not want to? | - If Yes; select A - If No; select B |
| 1. Where did you seek/receive help? | - If you did not seek any help; select A - If you went to a health facility; select B - If you went to the Police; select C - If you went to the Local administration; select D - If you went to the Child welfare; select E - If None of the above; select F |
