# Supplementary material for: Clustering of health risk behaviors among adolescents in Kilifi, Kenya, a rural Sub-Saharan African setting
Source: PLoS One. 2020 Nov 12;15(11):e0242186. doi: 10.1371/journal.pone.0242186 (PMC7660520; doi:10.1371/journal.pone.0242186)
Supplement: S2 Questionnaire — (DOCX) [file pone.0242186.s003.docx]

**YOUNG PEOPLE HEALTH SURVEY QUESTIONNARE (GIRIYAMA STUDIO VERSION)**

| ***Tafadhali vula virahu na nguo nyingine here, jaketi,sweta pulova, ili nidime kukupima kilo na ure)*** | | | |
| --- | --- | --- | --- |
| Ure | [_][_][_].[_] cm | | |
| Walamu wa mkono | [_][_] . [_]cm | | |
| Uziho | [_][_] . [_]kg | | |
| 1. Vidze unashoma skuli? | - Kala ni eeeh tsagula A - Kala ni laa tsagula B | | |
| 1. Uu/klasi cha nyingahi/fomu nyingahi? | - Kala ukilasi cha 3;tsagula A - Kala ukilasi cha 4;tsagula B - Kala ukilasi cha 5; tsagula C - Kala ukilasi cha 6; tsagula D - Kala ukilasi cha 7; tsagula E - Kala ukilasi cha 8; tsagula F - Kala ufomu 1; tsagula G - KalaF ufomu 2; tsagula H - Kala ufomu 3; tsagula I - Kala ufomu 4; tsagula J - Kala ucollege; tsagula K - Kala ni nyingine tsagula L na ueleze. | | |
| 1. Wakathi wa mwisho urekala kufahiwa wapata usaidhizi hiko? | - Kala kumalire usaidhizi; tsagula A - Kala wakwenda kwa spitali ya serikali; tsagula B - Kala wakwenda kwa spitali ya kibanfsi; tsagula C - Kala wakwenda kwa dawa za dukani; tsagula D - Kala wakwenda kwa dhuka ra madawa; tsagula E - Kala wakwenda kwa muganga wa kienyeji;tsagula F - Kala wakwenda kutu kungine; tsagula G | | |
| **Maswali 4 gathuwago ndakuza kuhusu usafi wa meno na kuoga mikono.** | | | |
| 1. Kwa zi siku 30 zidzekira, ni mara nyingahi kwa siku uretsula meno ama kupiga mswaki ? | - Kala kutsulire ama kupiga mswaki meno kwa siku 30 zidzekira. Tsagula A - Kala ni tsini ya mara mwenga kwa siku; tsagula B - Kala ni mara 1 kwa siku;tsagula C - Kala ni mara 2 kwa siku; tsagula D - Kala ni mara 3 kwa siku; tsagula E - Kala ni mara 4 ama zaidhi kwa siku; tsagula F | | |
| 1. Kwa zo siku 30 zidzekira, waoga mikono mara nyingahi kabila kurya? | - Kala ni hatha; tsagula A - Kala si sana; tsagula B - Kala ni mara nyingine; tsagula C - Kala ni mara nyinji; tsagula D - Kala ni kila mara; tsagula E | | |
| 1. Kwa zo siku 30 zidzekira,wahumira sabuni mara nyingahi urekala unaoga mikono baada ya kwenda chooni? | - Kala ni hatha; tsagula A - Kala si sana; tsagula B - Kala ni mara nyingine; tsagula C - Kala ni mara nyinji; tsagula D - Kala ni kila mara; tsagula E | | |
| 1. Kwa zo siku 30 zidzekira, ni mara nyingahi urehumira sabuni wakathi ureoga mikono? | - Kala ni hatha; tsagula A - Kala si sana; tsagula B - Kala ni mara nyingine; tsagula C - Kala ni mara nyinji; tsagula D - Kala ni kila mara; tsagula E | | |
|  |  | | |
| **Maswali 5 gathuwago ndakuza kuhusu udimazho kurya ama kunwa.** | | | |
| 1. Kwa zo siku 30 zidzekira,je ni mara nyingahi urekala na nzala kwa sababu kakuna chakurya cha kuthosha mudzini? | - Kala ni hatha;tsagula A - Kala si sana;tsagula B - Kala ni mara nyingine;tsagula C - Kala ni mara nyinji;tsagula D - Kala ni kila mara;tsagula E | |  |
| 1. Kwa zo siku 30 zidzekira, ni mara nyingahi kwa siku urerya matunda,here machungwa, mapapayu,mananasi,maembe,nazi,kunazi,mapera,makapu,mtsanganyiko wa matunda? | - Kala kurirye tunda kwa siku 30 zidzizokira;tsagula A - Kala ni tsini ya mara mwenga kwa siku;tsagula B - Kala ni mara 1 kwa siku;tsagula C - Kala ni mara 2 kwa siku;tsagula D - Kala ni mara 3 kwa siku’tsagula E - Kala ni mara 4 kwa siku;tsagula F - Kala ni mara 5 ama zaidhi ya zizo kwa siku;tsagula G | |  |
| 1. Kwa zo siku 30 zidzekira,ni mara nyingahi kwa siku urerya mboga here, sukuma wiki,karotsi,kisenywa,mnavu,kikosho? | - Kala kurirye mboga kwa siku 30 zidzekira;tsagula A - Kala ni tsini ya mara mwenga kwa siku;tsagula B - Kala ni mara 1 kwa siku;tsagula C - Kala ni mara 2 kwa siku;tsagula D - Kala ni mara 3 kwa siku;tsagula E - Kala ni mara 4 kwa siku;tsagula F - Kala ni mara 5 ama zaidhi ya zizo kwa siku;tsagula G | |  |
| 1. Kwa zo siku 30 zidzekira,ni mara nyingahi kwa siku urenwa kinywaji here soda? | - Kala kunwire soda kwa siku 30 zidzekira;tsagula A - Kala ni tsini ya mara 1 kwa siku;tsagula B - Kala ni mara 1 kwa siku;tsagula C - Kala ni mara 2 kwa siku;tsagula D - Kala ni mara 3 kwa siku;tsagula E - Kala ni mara 4 kwa siku;tsagula F - Kala ni mara 5 ama zaidhi ya zizo kwa siku;tsagula G | |  |
| 1. Kwa zo siku 7 zidzekira, ni kwa siku nyingahi urerya chakurya kumbola kwa kibanda here, chips, virazi zha kukalanga, mahamuri, chapati? | - Kala ni siku 0;tsagula A - Kala ni siku 1;tsagula B - Kala ni siku 2;tsagula C - Kala ni siku 3;tsagula D - Kala ni siku 4;tsagula E - Kala ni siku 5;tsagula F - Kala ni siku 6;tsagula G - Kala ni siku 7;tsagula H | |  |
| **Maswali 4 gathuwago, nindakuza kuhusu kunwa uchi. Ii inamanisha kunwa mnazi,changaa, bia. Kunwa uchi kaimanisha kuhenda kuthatha vichache here divai. Kinywadzi inamanisha gilasi,thupa ya bia,gilasi thithe ya uchi ama mtsanganyiko.** | | | |
| 1. Wakala una miaka mingahi urenwa uchi kwa mara ya kwanza,si kuthatha vichache? | - Kala kudzangwe kunwa uchi kamare,isokala kuthatha thu; tsagula A - Kala ni zaidhi ama tsini ya miaka 7;tsagula B - Kala ni miaka 8 ama 9; tsagula C - Kala ni miaka 10 ama 11;tsagula D - Kala ni miaka 12 ama 13;tsagula E - Kala ni miaka 14 ama 15;tsagula F - Kala ni miaka 16 ama 17;tsagula G - Kala ni miaka 18 ama zaidhi;tsagula H | | |
| 1. Kwa zo siku 30 zidzekira, ni siku nyingahi urepata angalau kinwadzi kimwenga cha uchi? | - Kala ni siku 0;tsagula A - Kala ni siku 1 tha 2;tsagula B - Kala ni siku 3 tha 5;tsagula C - Kala ni siku 6 tha 9;tsagula D - Kala ni siku 10 tha 19; tsagula E - Kala ni siku 20 tha 29;tsagula F - Kala ni siku zosi 30;tsagula G | |  |
| 1. Kwa zo siku 30 zidzekira, zo siku urenwa uchi, wanwa vinywadzi kwa siku? | - Kala kunwirwe uchi kwa siku 30 zidzekira;tsagula A - Kala ni tsini ya kinwadzi 1;tsagula B - Kala ni kinwadzi 1;tsagulaC - Kala ni vinwadzi 2;tsagula D - Kala ni vinwadzi 3;tsagula E - Kala ni vinwadzi 4;tsagula F - Kala ni vinwadzi 5 ama zaidhi;tsagula G | |  |
| 1. Kwa zo siku 30 zidzekira,je waupatadze wo uchi urenwa*?(Tsagula jibu mwenga hakeye)* | - Kala kunwire uchi kwa siku 30 zidzekira;tsagulaA - Kala wagula kwa stoo,duka ama kwa mchuuzi;tsagula B - Kala wampa mtu mungine pesa akugulire;tsagula C - Kala wapata kumbola kwa marafikizo;tsagula D - Kala wapata kula kwa familiyo;tsagula E - Kala waiyaama kuhala bila ruhusa;tsagula F - Kala waupata kwa ngira nyingine;tsagula G | |  |
| **Kutatalika-tatalika wakathi wakwanenda, kutsodima kunena tototo, kuhahika ni dalili za kunyesa udzareya.** | | | |
| 1. Wakathi wa maishago, ni mara nyingahi urenwa uchi tha ukidzisikira udzareya? | - Kala ni mara 0;tsagula A - Kala ni mara 1 ama 2;tsagula B - Kala ni mara 3 ama 9;tsagula C - Kala ni mara10 ama zaidhi;tsagula D |  | |
| 1. Wakathi wa maishago,ni mara nyingahi uredzipata matatani na familia yo ama marafikigo, kukosa kwenda skuli, ama kupigana kwa sababu ya urevi? | - Kala ni mara 0;tsagula A - Kala ni mara 1 ama 2;tsagula B - Kala ni mara 3 ama 9;tsagula C - Kala ni mara 10 ama zaidhi;tsagula D |  | |
| **Maswali 3 gathuwago ndakuza kuhusu mahumizi ga dawa za kurezha. Zizi ni here bhangi,amphetamines, unga,za kunusa.** | | | |
| 1. Were unamiaka mingahi wakathi urehumira dawa za kurezha kwa mara ya kwanza? | - Kala kudzangwe kuhumira madawa ga kurezha;tsagula A - Kala ni miaka 7 ama tsiniye;tsagula B - Kala ni miaka 8 ama 9;tsagula C - Kala ni miaka 10 ama 11;tsagula D - Kala ni miaka 12 ama 13;tsagula E - Kala ni miaka 14 ama 15;tsagula F - Kala ni miaka 16 ama 17;tsagula G - Kala ni miaka 18 ama zaidhi;tsagula H |  | |
| 1. Wakathi wa maishago,je ni mara nyingahi urehumira marijuana(Bangi,boza,bomu dom,holy herb) | - Kala ni mara 0;tsagula A - Kala ni mara 1 ama 2;tsagula B - Kala ni mara 3 hadhi 9;tsagula C - Kala ni mara 10 hadhi 19;tsagula D - Kala ni mara 20 ama zaidhi;tsagula E |  | |
| 1. Wakathi wa maishago ni mara ningahi urenusa gluu? | - Kala ni mara 0;tsagula A - Kala ni mara 1 ama 2;tsagula B - Kala ni mara 3 hadhi 9;tsagula C - Kala ni mara 10 hadhi 19;tsagula D - Kala ni mara 20 ama zaidhi;tsagula E |  | |
| **Maswali gathuwago ganauza kuhusu udzizhokala ukidzisikira wiki mbiri zirekira. Tafadhali piga tiki kwenye kibox kiricho hehi na urizhosikira. (Major Depression Inventory)** | | | |
| 1. Vidze wakala na kubandika moyo ama kuhuzunika? | - Kala kahalire na wakathi dza uo;tsagula A - Kala ni kwa wakathi mungine;tsagula B - Kala ni tsini kidogo ya nusu ya wakathi;tsagula C - Kala ni dzulu kidogo ya nusu ya wakathi;tsagula D - Kala ni wakathi munji;tsagula E - Kala ni wakathi wosi; tsagula F | | |
| 1. Vidze wangamiza hamu kwa mautugo ga kila siku? | - Kala kahalire na wakathi dza uo;tsagula A - Kala ni kwa wakathi mungine;tsagula B - Kala ni tsini kidogo ya nusu ya wakathi;tsagula C - Kala ni dzulu kidogo ya nusu ya wakathi;tsagula D - Kala ni wakathi munji;tsagula E - Kala ni wakathi wosi; tsagula F | | |
| 1. Vidze wasikira kukosa nguvu | - Kala kahalire na wakathi dza uo;tsagula A - Kala ni kwa wakathi mungine;tsagula B - Kala ni tsini kidogo ya nusu ya wakathi;tsagula C - Kala ni dzulu kidogo ya nusu ya wakathi;tsagula D - Kala ni wakathi munji;tsagula E - Kala ni wakathi wosi; tsagula F | | |
| 1. Vidze wadzisikira kukosa ujasiri? | - Kala kahalire na wakathi dza uo;tsagula A - Kala ni kwa wakathi mungine;tsagula B - Kala ni tsini kidogo ya nusu ya wakathi;tsagula C - Kala ni dzulu kidogo ya nusu ya wakathi;tsagula D - Kala ni wakathi munji;tsagula E - Kala ni wakathi wosi; tsagula F | | |
| 1. Vidze wakala na kukosa muelekeo ama kuhukumika? | - Kala kahalire na wakathi dza uo;tsagula A - Kala ni kwa wakathi mungine;tsagula B - Kala ni tsini kidogo ya nusu ya wakathi;tsagula C - Kala ni dzulu kidogo ya nusu ya wakathi;tsagula D - Kala ni wakathi munji;tsagula E - Kala ni wakathi wosi; tsagula F | | |
| 1. Vidze udzangwe kudzisikira kukala maisha si muhimu kuishi? | - Kala kahana wakathi dza uo;tsagula A - Kala ni kwa wakathi mungine;tsagula B - Kala ni tsini kidogo ya nusu ya wakathi;tsagula C - Kala ni dzulu kidogo ya nusu ya wakathi;tsagula D - Kala ni wakathi munji;tsagula E - Kala ni wakathi wosi;tsagula F | | |
| 1. Vidze udzangwe kukala na shidha ya kumakinika here Kala unashoma gazeti ama kulola televisheni? | - Kala kahana wakathi dza uo;tsagula A - Kala ni kwa wakathi mungine;tsagula B - Kala ni tsini kidogo ya nusu ya wakathi;tsagula C - Kala ni dzulu kidogo ya nusu ya wakathi;tsagula D - Kala ni wakathi munji;tsagula E - Kala ni wakathi wosi;tsagula F | | |
| 1. Vidze udzangwe kudzisikira na wasiwasi? | - Kala kahana wakathi dza uo;tsagula A - Kala ni kwa wakathi mungine;tsagula B - Kala ni tsini kidogo ya nusu ya wakathi;tsagula C - Kala ni dzulu kidogo ya nusu ya wakathi;tsagula D - Kala ni wakathi munji;tsagula E - Kala ni wakathi wosi;tsagula F | | |
| 1. Vidze udzangwe kudzisikira kuujwa nyuma ama kushushwa? | - Kala kahana wakathi dza uo;tsagula A - Kala ni kwa wakathi mungine;tsagula B - Kala ni tsini kidogo ya nusu ya wakathi;tsagula C - Kala ni dzulu kidogo ya nusu ya wakathi;tsagula D - Kala ni wakathi munji;tsagula E - Kala ni wakathi wosi;tsagula F | | |
| 1. Vidze udzangwe kukala na shidha ya kulala usiku? | - Kala kahana wakathi dza uo;tsagula A - Kala ni kwa wakathi mungine;tsagula B - Kala ni tsini kidogo ya nusu ya wakathi;tsagula C - Kala ni dzulu kidogo ya nusu ya wakathi;tsagula D - Kala ni wakathi munji;tsagula E - Kala ni wakathi wosi;tsagula F | | |
| 1. Vidze udzangwe kupata shidha ya kukosa hamu ya kurya? | - Kala kahana wakathi dza uo;tsagula A - Kala ni kwa wakathi mungine;tsagula B - Kala ni tsini kidogo ya nusu ya wakathi;tsagula C - Kala ni dzulu kidogo ya nusu ya wakathi;tsagula D - Kala ni wakathi munji;tsagula E - Kala ni wakathi wosi;tsagula F | | |
| 1. Vidze udzangwe kupata kungezeka kwa hamu ya chakurya? | - Kala kahana wakathi dza uo;tsagula A - Kala ni kwa wakathi mungine;tsagula B - Kala ni tsini kidogo ya nusu ya wakathi;tsagula C - Kala ni dzulu kidogo ya nusu ya wakathi;tsagula D - Kala ni wakathi munji;tsagula E - Kala ni wakathi wosi;tsagula F | | |
| **Maswali 3 gathuwago ganauza kuhusu mazoezi ga mwiri. Mazoezi ga mwiri ni mazoezi ambago ganangeza mpigo wa moyo, na kuhenda kupumua tototo. Mazoezi ga mwiri ganadima kuhendwa kwa mizazigo, kuzaziga na marafiki,kutsembera kwenda skuli. Mifano ya mazoezi ga mwri ni kuuka malo,kuyera,kuhirika baskili, kuvina,kuzaziga mpira,kuogerera, mipra wa mikono.(Mahusikao ni atuu mendao skuli)** | | | |
| 1. Kwa siku 7 zidzekira,ni kwa siku nyingahi urekala mchangamfu wa mwiri kwa muda wa dakika 60 kwa siku? | - Kwa siku 0;tsagula A - Kwa siku 1;tsagula B - Kwa siku 2;tsagula C - Kwa siku 3;tsagula D - Kwa siku 4;tsagula E - Kwa siku 5;tsagula F - Kwa siku 6;tsagula G - Kwa siku 7;tsagula H | | |
| 1. Kwa siku 7 zidzekira,ni kwa siku nyingahi uretsembera ama kuhirika baskili kwenda ama kuuya kumbola skuli? | - Kwa siku 0;tsagula A - Kwa siku 1;tsagula B - Kwa siku 2;tsagula C - Kwa siku 3;tsagula D - Kwa siku 4;tsagula E - Kwa siku 5;tsagula F - Kwa siku 6;tsagula G - Kwa siku 7;tsagula H | | |
| 1. Kwa mwaka uu wa skuli, ni kwa siku nyingahi urekwenda kwa kilasi cha mazoezi ga mwiri kila wiki? | - Kwa siku 0;tsagula A - Kwa siku 1;tsagula B - Kwa siku 2;tsagula C - Kwa siku 3;tsagula D - Kwa siku 4;tsagula E - Kwa siku 5 ama zaidhi ya zizi;tsagula F | | |
| **Maswali gathuwago ganauza kuhusu wakathi uhumirao kukelesi kala kuskuli ama kuhenda kazi nyumbani.(Ahusiki ni ahoho mendao skuli)** | | | |
| 1. Ni muda wani uhunirao wakathi wa siku ya kawaida kukelesi na kulola televisheni, kunena na marafiki ama kala ukelesi unatsuha ngano. | - Kala ni tsini ya saa 1 kwa siku;tsagula A - Kala ni saa 1 hadhi 2 kwa siku;tsagula B - Kala ni masaa 3 hadhi 4 kwa siku;tsagula C - Kala ni masaa 5 hadhi 6 kwa siku;tsagula D - Kala ni masaa 7 hadhi 8 kwa siku;tsagula E - Kala ni zaidhi ya masaa 8 kwa siku;tsagula F | | |
| **Maswali 6 gathuwago ganauza kuhusu ujuzi/uzoefu skuli na nyumbani** | | | |
| 1. Kwa siku 30 zidzekira,ni kwa siku nyingahi urekosa vilasi ama kukosa kwenda skuli bila ruhusa? | - Kwa siku 0;tsagula A - Kwa siku 1 ama 2;tsagula B - Kwa siku 3 ama 5;tsagula C - Kwa siku 6 ama 9;tsagula D - Kwa siku 10 ama zaidhi;tsagula E | | |
| 1. Kwa siku 30 zidzekira, ni kwa mara nyingahi ahoho a skuli makala na usaidhizi na adzo kwako? | - Kala hatha;tsagula A - Kala si sana;tsagula B - Kala ni kwa wakathi mungine;tsagula C - Kala ni kwa wakathi munji;tsagula D - Kala ni kila mara; tsagula E | | |
| 1. Kwa siku 30 zidzekira, ni kwa mara nyingahi azhazio ama mwimirizio arelola kala udzatengeza kazi ya skuli. | - Kala hatha;tsagula A - Kala si sana;tsagula B - Kala ni kwa wakathi mungine;tsagula C - Kala ni kwa wakathi munji;tsagula D - Kala ni kila mara; tsagula E - Kala ni kila mara;tsagula E | | |
| 1. Kwa siku 30 zidzekira, ni kwa mara nyingahi azhazio ama mwimirizio makuelewa wasiwasio na shidhazo? | - Kala hatha;tsagula A - Kala si sana;tsagula B - Kala ni kwa wakathi mungine;tsagula C - Kala ni kwa wakathi munji;tsagula D - Kala ni kila mara; tsagula E | | |
| 1. Kwa siku 30 zidzekira, ni mara kwa nyingahi azhazio ama mwimirizio mamanya urizhokala unahenda na wakathiwo huru? | - Kala hatha;tsagula A - Kala si sana;tsagula B - Kala ni kwa wakathi mungine;tsagula C - Kala ni kwa wakathi munji;tsagula D - Kala ni kila mara; tsagula E | | |
| 1. Kwa siku 30 zidzekira, ni mara kwa nyingahi azhazio ama mwimirizio malola vituzho bila ruhusayo? | - Kala hatha;tsagula A - Kala si sana;tsagula B - Kala ni kwa wakathi mungine;tsagula C - Kala ni kwa wakathi munji;tsagula D - Kala ni kila mara; tsagula E | | |
| **Maswali 6 gathuwago ganauza kuhusu sigara na mahumizi ga kumbaku.** | | | |
| 1. Were una umri wani urejeza kuvuha sigara kwa mara ya kwanza? | - Kala kudzangwe kuvuha sigara;tsagula A - Kala ni miaka 7 ama tsinize;tsagula B - Kala ni miaka 8 ama 9;tsagula C - Kala ni miaka 10 ama 11;tsagula D - Kala ni miaka 12 ama 13;tsagula E - Kala ni miaka 14 ama 15;tsagula F - Kala ni miaka 16 ama 17;tsagula G - Kala ni miaka 18 ama zaidhi;tsagula H | | |
| 1. Kwa siku 30 zidzekira, ni kwa siku nyingahi udzevuha sigara? | - Kala ni siku 0;tsagula A - Kala ni siku 1 ama 2;tsagula B - Kala ni siku 3 hadhi 5;tsagula C - Kala ni siku 6 hadhi 9;tsagula D - Kala ni siku 10 hadhi 19;tsagula E - Kala ni siku 20 hadhi 29;tsagula F - Kala ni siku zosini 30;tsagula G | | |
| 1. Kwa siku 30 zidzekira, ni kwa siku nyingahi udzehumira vitu zha kumbaku,si sigara here toza? | - Kala ni siku 0;tsagula A - Kala ni siku 1 hadhi 2;tsagula B - Kala ni siku 3 hadhi 5;tsagula C - Kala ni siku 6 hadhi 9;tsagula D - Kala ni siku 10 hadhi 19;tsagula E - Kala ni siku 20 hadhi 29;tsagula F - Kala ni siku zosi 30;tsagula G | | |
| 1. Kwa muda wa miezi 12 idzekira,je wajeza kuricha kuvuha sigara? | - Kala kudzangwe kuvuha sigara;tsagula A - Kala kuvuhire sigara kwa miezi 12 idzekira;tsagula B - Kala ni eeeh;tsagula C - Kala ni hatha;tsagula D | | |
| 1. Kwa siku 7 zidzekira ni kwa siku nyingahi atu madzevuha mberezo? | - Kwa siku 0;tsagula A - Kwa siku 1 ama 2;tsagula B - Kwa siku 3 ama 4;tsagula C - Kwa siku 5 ama 6;tsagula D - Kwa siku zosi 7;tsagula E | | |
| 1. Ni hio kahi ya azhazio ama aiimirizio mahumirao kumbaku rorosi? | - Kala kahana;tsagula A - Kala ni babayo ama mwimirizio wa kilume;tsagula B - Kala ni mameyo ama mwimirizio wa kiche;tsagula C - Kala ni osini;tsagula D - Kala kumanya;tsagula E | | |
| **Maswali gathuwago ganauza kuhusu kupigana/kuheha. Kupigana nikala anafunzi aiiri amarika na nguvu sawa manaheha.** | | | |
| 1. Kwa muda wa miezi 12 idzekira, ni mara nyingahi urepigana/ureheha? | - Kala ni mara 0;tsagula A - Kala ni mara 1;tsagula B - Kala ni mara 2 ama 3;tsagula C - Kala ni mara 4 ama 5;tsagula D - Kala ni mara 6 ama 7;tsagula E - Kala ni mara 8 ama 9;tsagula F - Kala ni mara 10 ama 11;tsagula G - Kala ni mara 12 na zaidhi;tsagula H | | |
| Maswali 3 gathuwago ganauza kuhusu majeruhi mabaya garekupata. Jeruhi ni baya kala rindakuhenda ukose shuhulizo za siku nzima( here skuli, michezo ama kazi) ama inahenza kutibiwa ni dakitari ama nurse. | | | |
| 1. Kwa miezi 12 idzekira, ni mara nyingahi urejeruhiwa vibaya? | - Kala ni mara 0;tsagula A - Kala ni mara 1;tsagula B - Kala ni mara 2 ama 3;tsagula C - Kala ni mara 4 ama 5;tsagula D - Kala ni mara 6 ama 7;tsagula E - Kala ni mara 8 ama 9;tsagula F - Kala ni mara 10 ama 11;tsagula G - Kala ni mara 12 na zaidhi;tsagula H | | |
| 1. Kwa miezi 12 idzekira, ni jeruhi rani kali zadi ureripata? | - Kala kujeruhiwirwe sana kwa miezi 12 idzekira;tsagula A - Kala wabandika msoza na kuhemuka;tsagula B - Kala wapata kironda cha kutsinzwa ama kudungwa;tsagula C - Kala wapata jeraha ra singo na kitswa, nagongwa ukikala kudima kusoha;tsagula D - Kala wapata jeraha ra risasi;tsagula E - Kala washa vii-vii;tsagula F - Kala wagizirwa shumu ama warya dawa nyinji;tsagula G - Kala walumira na utu ungine;tsagula H | | |
| 1. Kwa miezi 12 idzekira, ni sababu yani iresababisha ro jeraha kali kwako? | - Kala kulumirire kwa miezi 12 idzekira;tsagula A - Kala wapata ajali ya gari ama wagongwa ni gari;tsagula B - Kala wagwa;tsaggula C - Kala wagwererwa ni kitu ama chakugonga;tsagula D - Kala wavamiwa ama kuhukanwa ama kupigana na mtu;tsagula E - Kala wakala kwa moho,karibu na moho ama kitu cha moho;tsagula F - Kala wanusa ama kumiza kitu kibaya;tsagula G - Kala jeraha rasababishwa ni kitu kingine;tsagula H | | |
| **Maswali mairi gathuwago ganauza kuhusu kuteswa/kubujwa. Kutsewa kunadza kala mwanafunzi ama kikundi cha anafunzi mananena,kuhenda mautu mai-mai kwa mwanziwao. Ni kutesa ikikala mwafunzi andadadishwa sana na mautu ambago si madzo,ama arichwe nze ya mautu kwa makusudi. Si kutesa ikalaho anafunzi enye nguvu sawa mandanenezana ama kupigana ama kutaniana kwa kirafiki.** | | | |
| 1. Kwa siku 30 zidzekira, ni kwa siku nyingahi urebuliwa/kuteswa/kubunjwa? | - Kala ni siku 0;tsagula A - Kala ni siku 1 ama 2;tasgula B - Kala ni siku 3 hadhi 5;tsagula C - Kala ni siku 6 hadhi 9;tsagula D - Kala ni siku 10 hadhi 19;tsagula E - Kala ni siku 20 hadhi 29;tsagula F - Kala ni siku sozi 30;tsagula G | | |
| 1. Kwa siku 30 zidzekira, wabuliwa/wateswa sana kihizho? | - Kala kuteserwe kwa siku 30 sidzekira;tsagula A - Kala wagongwa,ukipigwa,ukisukumwa ama ukifungirwa ndani;tsagula B - Kala watsekwa kwa sababu ya kabila,urai na rangi;tsagula C - Kala wahenderwa mzaha kwa sababu ya diniyo;tsagula D - Kala wahenzderwa mzaha wa ngono,kimaneno na kivihendo;tsagula E - Kala wathengwa kwa michezo kimakusudi,ama kutsorungwa kabisa;tsagule F - Kala wahenderwa mzaha kwa sababu ya mwirio ama uso wo zho urizho;tsagula G - Kala wateswa kwa ngira nyingine;tsagule H | | |
| **Vikara nindahenza nikuze kuhusu mautu ga kuhala.** | | | |
| 1. Vidze vino wahala/wahalwa? | - Kala wahala/wahalwa;tsagule A - Kala kudzangwe kuhala/kuhalwa lakini unaishi na mtu jinsia nyingine;tsagula B - Kala wafererwa;tsagula C - Kala mwarichana;tsagula D - Kala mwarichana kwa sababu were kamuelewana na mulumeo/mucheo;tsagula E - Kala kudzangwe kuhala/kuhalwa;tsagule F | | |
| 1. Ho unahala/kuhalwa ho kwanza were una umri wani? | - Kala ni tsini ya miaka 13 ama haho;tsagula A - Kala ni miaka 14;tsagula B - Kala ni miaka 15;tsagula C - Kala ni miaka 16;tsagula D - Kala ni miaka 17;tsagula E - Miaka 18 ama zaidhi;tsagula F - Kala kumanya;tsagula G | | |
| **Kuenderera, nehenza fuzungumze kuhusu mimba/Uja uziho.** | | | |
| 1. Ni mara nyingahi udzekala na mimba/uja uziho ama kugwiza mimba? | - Kala ni mara 0;tsagula A - Kala ni mara 1;tsagula B - Kala ni mara 2 ama zaidhi;tsagula C - Kala kumanya;tsagula D | | |
| 1. Were una miaka mingahi ho urepata mimba/uja uziho mara ya kwanza(Ache hakeye) | - Kala ni tsini ya ama miaka 13;tsagula A - Kala ni miaka 14;tsagula B - Kala ni miaka 15;tsagula C - Kala ni miaka 16;tsagula D - Kala ni miaka 17;tsagula E - Kala miaka 18 ama zaidhi;tsagula F - Kala kumanya;tsagula G | | |
| 1. Yo mimba/uja uziho zha anzadze? | - Kala wazhala mwana mzima; tsagula A - Kala uchere na mimba;tsagula B - Kala walazha;tsagula C - Kala wamboza ama yamwagika;tsagula D - Kala kumanya;tsagula E | | |

| Ii sehemu ya mwisho ni kuhusu tabia za ngono | |
| --- | --- |
| 1. Vidze wawahi kulala na mulume/muche? | - Kala ni eeeh;tsagula A - Kala ni hatha;tsagula B |
| 1. Were una miaka mingahi ho urelala na mulume/muche? | - Kala kudzangwe kuhenda ngono na mtu;tsagula A - Kala were una miaka 11 ama tsinize;tsagula B - Kala were una miaka 12;tsagula C - Kala were una miaka 13;tsagula D - Kala were una miaka 14;tsagula E - Kala were una miaka 15;tsagula F - Kala were una miaka 16 ama 17;tsagula G - Kala were una miaka 18 ama zaidhi;tsagula H |
| 1. Wakathi wa maishago, wawahi kulala na alume/ache angahi? | - Kala kudzangwe kuhenda ngono;tsagula A - Kala ni mtu 1;tsagula B - Kala ni atu 2;tsagula C - Kala ni atu 3;tsagula D - Kala ni atu 4;tsagula E - Kala ni atu 5;tsagula F - Kala ni atu 6 ama zaidhi;tsagula G |
| 1. Wakathi wa mwisho kulala na mulume/muche wahumira mpira(Kondomu)? | - Kala kudzangwe kuhenda ngono;tsagula A - Kala ni eeeh;tsagula B - Kala ni hatha;tsagula C |
| 1. Wakathi wa mwisho kuhenda ngono,je we ama ye rafikiyo mwahumira mautu ga kuzulia mimba,here kumboza nze, tembe za kuzulia mimba, ama ngira nyingine yoyosi ya kuzulia mimba? | - Kala kudzangwe kuhenda ngono;tsagula A - Kala ni eeeh;tsagula B - Kala ni hatha;tsagula C - Kala kumanya;tsagula D |
| 1. Vidze wawahi kukengwa ili uhende ngono/ulale na mtu jaho were kwenzi? | - Kala ni eeeh;tsagula A - Kala ni hatha;tsagula B |
| 1. Vidze wawahi kufungirwa kwenye chumba ili uhende ngono/ulale na mtu jaho were kwenzi? | - Kala ni eeeh;tsagula A - Kala ni hatha;tsagula B |
| 1. Je wawahi kulazimishwa kuhenda ngono/kulala na mtu jaho were kwenzi kuvihenda? | - Kala ni eeeh;tsagula A - B. Kala ni hatha;tsagula B |
| 1. Je watafuta/wapata usaidhizi hiko? | - Kala kumalire usaidhizi wowosi;tsagula A - Kala wakwenda kituo cha afya;tsagula B - Kala kwakwenda polisi;tsagula C - Kala wakwenda serikali ya mtaa;tsagula D - Kala wakwenda kwa idara ya ahoho;tsagula E - Kala kahana idzehadzwa;tsagula F |
