# Supplementary material for: Clustering of health risk behaviors among adolescents in Kilifi, Kenya, a rural Sub-Saharan African setting
Source: PLoS One. 2020 Nov 12;15(11):e0242186. doi: 10.1371/journal.pone.0242186 (PMC7660520; doi:10.1371/journal.pone.0242186)
Supplement: S3 Questionnaire — (DOC) [file pone.0242186.s004.doc]

**YOUNG PEOPLE HEALTH SURVEY QUESTIONNARE (SWAHILI STUDIO VERSION)**

| **Anthropometric measurements**  ***(Tafadhali vua viatu vyako,na nguo za ziada kama jaketi,koti,sweta pulova ili tuweze kupima uzito na urefu)*** | | |
| --- | --- | --- |
| Urefu | [_][_][_].[_] cm | |
| Upana wa mkono | [_][_] . [_]cm | |
| Uzito | [_][_] . [_]kg | |
| 1. Je uko shule? | - Kama ni ndio Bonyeza A - Kama ni la Bonyeza B | |
| 1. Je uko daraja/darasa/kidato cha ngapi? | - Kama uko darasa la 3 Bonyeza A - Kama uko darasa la 4 Bonyeza B - Kama uko darasa la 5 Bonyeza C - Kama uko darasa la 6 Bonyeza D - Kama uko darasa la 7 Bonyeza E - Kama uko darasa la 8 Bonyeza F - Kama ukokidato cha1 Bonyeza G - Kama ukokidato cha 2 Bonyeza H - Kama ukokidato cha 3 Bonyeza I - Kama ukokidato cha 4 Bonyeza J - Kama uko College/Chuo kikuu Bonyeza K - Kama ni Nyengine eleza kwa kuBonyeza L | |
| 1. Mara ya mwisho ulivyokuwa mgonjwa/hujisikii vizuri, ulitafuta wapi usaidizi? | - kama ni hapana Bonyeza A - kama ni kituo cha serikal Bonyeza B - kama ni kituo cha kibinafsi Bonyeza C - kama ni kwa Dawa za dukani Bonyeza D - kama ni kwa duka la dawa Bonyeza E - kama ni kwa mtabibu wa kienyeji Bonyeza F - kama ni Kwengineko Bonyeza G | |
| **Maswali 4 yafuatayo yanauliza kuhusu kusafish meno na kuosha mikono.** | | |
| 1. Katika siku 30 zilizopita,je ni mara ngapi kwa siku umekuwa ukisafisha meno au kupiga mswaki? | - Kama hukusafisha meno/kupiga mswaki katika siku 30 zilizopita Bonyeza A - Kama ni chini ya mara 1 kwa siku Bonyeza B - Kama ni mara 1 kwa siku Bonyeza C - Kama ni mara 2 kwa siku Bonyeza D - Kama ni mara 3 kwa siku Bonyeza E - Kama ni mara 4 au zaidi kwa siku Bonyeza F | |
| 1. Katika siku 30 zilizopita,ni mara ngapi umekuwa ukiosha mikono kabla kutumia chakula? | - kama ni hapana hukuosha Bonyeza A - kama si sana Bonyeza B - kama ni mara nyingine Bonyeza C - kama ni mara nyingi Bonyeza D - kama ni kila wakati Bonyeza E | |
| 1. Katika siku 30 zilizopita,ni kwa mara ngapi umekuwa ukiosha mikono baada ya kutoka/kutumia choo? | - kama ni hapana hukuosha Bonyeza A - kama si sana Bonyeza B - kama ni mara nyingine Bonyeza C - kama ni mara nyingi Bonyeza D - kama ni kila wakati Bonyeza E | |
| 1. Katika siku 30 zilizopita,ni mara ngapi umetumia sabuni kuosha mikono? | - kama ni hapana-hukutumia sabuni Bonyeza A - kama si sana Bonyeza B - kama ni mara nyingine Bonyeza C - kama ni mara nyingi Bonyeza D - kama ni kila wakati Bonyeza E | |
| **Maswali 5 yafuatayo yanauliza unachokula na kunywa** | | |
| 1. Katika siku 30 zilizopita,ni mara ngapi umeenda njaa kwa sababu hakukuwa na chakula cha kutosha nyumbani? | - kama ni hapana Bonyeza A - kama si sana Bonyeza B - kama ni mara nyingine - mara nyingi Bonyeza D - kama ni kila wakati Bonyeza E |  |
| 1. Katika siku 30 zilizopita,ni mara ngapi kwa siku umekula tunda kama machungwa,papai,nanasi,maembe,nazi,kunazi,mapera,limau ua mchanganyiko wa matunda. | - Kama hukula tunda katika siku 30 zilizopita Bonyeza A - Kama ni chini ya mara 1 kwa siku Bonyeza B - Kama ni mara 1 kwa siku Bonyeza C - Kama ni mara 2 kwa siku Bonyeza D - Kama ni mara 3 kwa siku Bonyeza E - Kama ni mara 4 kwa siku Bonyeza F - Kama ni mara 5 au zaidi kwa siku Bonyeza G |  |
| 1. Katika siku 30 zilizopita,ni mara ngapi kwa siku umekuwa ukila mboga kama kabeji,sukuma wiki,karoti,mchicha,mnavu na mrenda. | - Kama hukula mboga katika siku 30 zilizopita Bonyeza A - Kama ni chini ya mara 1 kwa siku Bonyeza B - Kama ni mara 1 kwa siku Bonyeza C - Kama ni mara 2 kwa siku Bonyeza D - Kama ni mara 3 kwa siku Bonyeza E - Kama ni mara 4 kwa siku Bonyeza F - Kama ni mara 5 au zaidi kwa siku Bonyeza G |  |
| 1. Katika siku 30 zilizopita.ni mara ngapi kwa siku umekuwa ukinywa vinywaji kama soda | - Kama hukunywa kinywaji kama vile soda katika siku 30 zilizopita Bonyeza A - Kama ni chini ya mara 1 kwa siku Bonyeza B - Kama ni mara 1 kwa siku Bonyeza C - Kama ni mara 2 kwa siku Bonyeza D - Kama ni mara 3 kwa siku Bonyeza E - Kama ni mara 4 kwa siku Bonyeza F - Kama ni mara 5 au zaidi kwa siku Bonyeza G |  |
| 1. Katika siku 7 zilizopita,ni kwa siku ngapi umekula chakula kutoka kwa vibanda vya chakula kama chips,viazi karai,mahamri,chapati? | - kwa siku 0/bado Bonyeza A - kwa siku 1Bonyeza B - kwa siku 2 Bonyeza C - kwa siku 3 Bonyeza D - kwa siku 4 Bonyeza E - kwa siku 5 Bonyeza F - kwa siku 6 Bonyeza G - kwa siku 7 Bonyeza H |  |
| **Maswali 4 yafuatayo ni kuhusu unywaji pombe.Hii inajumulisha kunya mnazi, changaa, beer, pombe.Kunywa pombe haijumulishi kuonja/kunywa tama kidogo ya divai kwa ajili ya dini.Kinywaji ni glasi ya wine, chupa ya beer, glass ndogo ya pombe au mchanganyiko wa kinywaji.** | | |
| 1. Je ulikuwa na umri gani mara ya kwanza,ulipokunywa pombe/kileo mbali na kuonja kidogo. | - Kama hujawahi kutumia kinywaji cha pombe mbali na kuonja kidogo Bonyeza - Kama ni miaka 7 au chini yake Bonyeza B - Kama ni miaka 8 au 9 Bonyeza C - Kama ni miaka 10 au11 Bonyeza D - Kama ni miaka 12 au 13 Bonyeza E - Kama ni miaka 14 au 15 Bonyeza F - Kama ni miaka 16 au 17 - Kama ni miaka 18 au zaidi | |
| 1. Katika siku 30 zilizopita,ni siku ngapi ulipata kinywaji kimoja chenye pombe? | - kama ni siku 0/bado Bonyeza A - kama ni siku 1 au 2 Bonyeza B - kama ni siku 3 hadi 5 Bonyeza C - kama ni siku 6 hadi 9 Bonyeza D - kama ni siku 10 hadi 19 Bonyeza E - kama ni siku 20 hadi 29 Bonyeza F - kama ni siku zote 30 Bonyeza G |  |
| 1. Katika siku 30 zilizopita,katika siku ulizokunywa pombe,ni vinywaji vingapi umekuwa ukinywa kwa siku? | - Kama hukunywa pombe katika siku 30 zilizopita Bonyeza A - Kama ni chini ya kinywaji kimoja Bonyeza B - Kama ni kinywaji 1 Bonyeza C - Kama ni vinywaji 2 Bonyeza D - Kama ni vinywaji 3 Bonyeza E - Kama ni vinywaji 4 Bonyeza F - Kama ni vinywaji 5 au zaidi Bonyeza G |  |
| 1. Katika siku 30 zilizopita, ulipata vipi pombe uliyokunywa? (chagua jibu moja) | - Kama hukunywa pombe katika siku 30 zilizopita Bonyeza A - Kama ulinunua kwa duka au kwa mchuruzi Bonyeza B - Kama ulimpa mtu pesa na akaninunulia Bonyeza C - Kama ulipata kwa marafiki Bonyeza D - Kama ulipata kwa familia yako Bonyeza E - Kama uliiba/ulipata bila ruhusa Bonyeza F - Kama ulipata kwa njia nyengine Bonyeza G |  |
| **Kurega rega ukitembea,kutoongea vizuri,na kutapika ni ishara za kulewa sana** | | |
| 1. Katika maisha yako,ni mara ngapi ulikunywa pombe sana mpaka ukalewa kabisa? | - kama ni mara 0/bado Bonyeza A - mara 1 au zaidi Bonyeza B - kama ni mara 3 hadi 9 Bonyeza C - kama ni mara 10 au zaidi Bonyeza D |  |
| 1. Katika maisha yako,ni mara ngapi umepata shida na familia au marafiki,kukosa shule,umejipata wateta kwa sababu ya kunywa pombe? | - kama ni mara 0/bado Bonyeza A - mara 1 au zaidi Bonyeza B - kama ni mara 3 hadi 9 Bonyeza C - kama ni mara 10 au zaidi Bonyeza D |  |
| **Maswali 3yafuatayo ni kuhusu utumiaji wa madawa ya kulevya.**  **Hii inajumulisha marijuana, amphetamines, cocaine, na ya kunusa.** | | |
| 1. Je ulikuwa na umri gani ulipoanza kutumia madawa ya kulevya | - Kama hujawahi kutumia madawa ya kulevya Bonyeza A - Kama ni miaka 7 au chini Bonyeza B - Kama ni miaka 8 au 9 Bonyeza C - Kama ni miaka 10 hadi 11Bonyeza D - Kama ni miaka 12 au 13 Bonyeza E - Kama ni miaka 14 au 15 Bonyeza F - Kama ni miaka 16 au 17 Bonyeza G - Kama ni miaka 18 au zaidi Bonyeza H |  |
| 1. Katika maisha yako,ni mara ngapi umetumia marijuana(pia inaitwa bangi, bosa,bomu,dom,holy,herb,hashish) | - kama ni mara 0/bado Bonyeza A - kama ni mara 1 or 2 Bonyeza B - kama ni mara 3 hadi 9 Bonyeza C - kama ni mara 10 hadi 19 Bonyeza D - kama ni mara 20 au zaidi Bonyeza E |  |
| 1. Katika maisha yako,ni mara ngapi umenusa glu? | - kama ni mara 0/bado Bonyeza A - kama ni mara 1 au 2 Bonyeza B - kama ni mara 3 au 9 Bonyeza C - kama ni mara 10 au 19 Bonyeza D - kama ni mara 20 au zaidi Bonyeza E |  |
| **Maswali yafuatayo ni kuhusu vile umejisikia/umejihisi kwa wiki 2 zilizopita.**  **Weka kistari kwa kisanduku ambacho inakaribia na vile umejisikia/jihisi.(Major Depression Inventory(MDI)** | | |
| 1. Je umejisikia kuvunjika moyo au mwenye huzuni? | - - Kama haijatokea Bonyeza A   - Kama ni kwa wakati mwingine Bonyeza B   - Kama ni chini ya nusu ya wakati Bonyeza C   - Kama ni juu kidogo ya nusu ya wakati Bonyeza D   - Kama ni wakati mwingi Bonyeza E   - Kama ni wakati wote Bonyeza F | |
| 1. Je umepoteza hamu ya shughuli zako za kila siku? | - - Kama haijatokea Bonyeza A   - Kama kwa wakati mwingine Bonyeza B   - Kama ni chini ya nusu ya wakati Bonyeza C   - Kama ni juu kidogo ya nusu ya wakati Bonyeza D   - Kama ni kwa wakati mwingi Bonyeza E   - Kama ni wakati wote Bonyeza F | |
| 1. Je umejihisi kukosa nguvu? | - - Kama haijatokea Bonyeza A   - Kama kwa wakati mwingine Bonyeza B   - Kama ni chini ya nusu ya wakati Bonyeza C   - Kama ni juu kidogo ya nusu ya wakati Bonyeza D   - Kama ni kwa wakati mwingi Bonyeza E   - Kama ni wakati wote Bonyeza F | |
| 1. Je umejisikia huna ujasiri wa kutosha? | - - Kama haijatokea Bonyeza A   - Kama kwa wakati mwingine Bonyeza B   - Kama ni chini ya nusu ya wakati Bonyeza C   - Kama ni juu kidogo ya nusu ya wakati Bonyeza D   - Kama ni kwa wakati mwingi Bonyeza E   - Kama ni wakati wote Bonyeza F | |
| 1. Je umekuwa na fikra mbaya au kusikia kuhukumika? | - - Kama haijatokea Bonyeza A   - Kama kwa wakati mwingine Bonyeza B   - Kama ni chini ya nusu ya wakati Bonyeza C   - Kama ni juu kidogo ya nusu ya wakati Bonyeza D   - Kama ni kwa wakati mwingi Bonyeza E   - Kama ni wakati wote Bonyeza F | |
| 1. Je umejihisi kuwa hakuna umuhimu wa kuishi? | - - Kama haijatokea Bonyeza A   - Kama kwa wakati mwingine Bonyeza B   - Kama ni chini ya nusu ya wakati Bonyeza C   - Kama ni juu kidogo ya nusu ya wakati Bonyeza D   - Kama ni kwa wakati mwingi Bonyeza E   - Kama ni wakati wote Bonyeza F | |
| 1. Je umukuwa na ugumu wa kumakinika kwa mfano wakati ukusoma gazeti au kuangalia runinga? | - - Kama haijatokea Bonyeza A   - Kama kwa wakati mwingine Bonyeza B   - Kama ni chini ya nusu ya wakati Bonyeza C   - Kama ni juu kidogo ya nusu ya wakati Bonyeza D   - Kama ni kwa wakati mwingi Bonyeza E   - Kama ni wakati wote Bonyeza F | |
| 1. Je umejisikia wasiwasi? | - - Kama haijatokea Bonyeza A   - Kama kwa wakati mwingine Bonyeza B   - Kama ni chini ya nusu ya wakati Bonyeza C   - Kama ni juu kidogo ya nusu ya wakati Bonyeza D   - Kama ni kwa wakati mwingi Bonyeza E   - Kama ni wakati wote Bonyeza F | |
| 1. Je umejisia kushindwa au kushushwa? | - - Kama haijatokea Bonyeza A   - Kama kwa wakati mwingine Bonyeza B   - Kama ni chini ya nusu ya wakati Bonyeza C   - Kama ni juu kidogo ya nusu ya wakati Bonyeza D   - Kama ni kwa wakati mwingi Bonyeza E   - Kama ni wakati wote Bonyeza F | |
| 1. Je umekuwa ukipata shida ya kulala usiku? | - - Kama haijatokea Bonyeza A   - Kama kwa wakati mwingine Bonyeza B   - Kama ni chini ya nusu ya wakati Bonyeza C   - Kama ni juu kidogo ya nusu ya wakati Bonyeza D   - Kama ni kwa wakati mwingi Bonyeza E   - Kama ni wakati wote Bonyeza F | |
| 1. Je umekumbwa na upungufu wa hamu ya chakula? | - - Kama haijatokea Bonyeza A   - Kama kwa wakati mwingine Bonyeza B   - Kama ni chini ya nusu ya wakati Bonyeza C   - Kama ni juu kidogo ya nusu ya wakati Bonyeza D   - Kama ni kwa wakati mwingi Bonyeza E   - Kama ni wakati wote Bonyeza F | |
| 1. Je umekumbwa na ongezeko la hamu ya chakula? | - Kama haijatokea Bonyeza A - Kama kwa wakati mwingine Bonyeza B - Kama ni chini ya nusu ya wakati Bonyeza C - Kama ni juu kidogo ya nusu ya wakati Bonyeza D - Kama ni kwa wakati mwingi Bonyeza E - Kama ni wakati wote Bonyeza F | |
| **Maswali 3 yafuatayo yanauliza kuhusu mazoezi ya mwili.mazoezi ya mwili ni shuhuli ambazo huongeza pigo la moyo na kukufanya kupumua/kuhema kwa nguvu.Mazoezi yanaweza kufanyika kwa michezo,kucheza na marafiki au kutembea kwenda shule.Baadhi ya mifano ya mazoezi ya mwili ni kukimbia,kutembea haraka haraka,kuendesha baiskeli,kucheza dansi,kucheza mpira,kuogelea,mpira wa mkono.(kwa vijana wanaenda shule)** | | |
| 1. Katika siku 7 zilizopita,ni mara ngapi umekuwa mchangamfu kimaumbile kwa jumla ya dakika 60 kwa siku? | - siku 0/bado Bonyeza A - siku 1 Bonyeza B - siku 2 Bonyeza C - siku 3 Bonyeza D - kwa siku 4 Bonyeza E - siku 5 Bonyeza F - siku 6 Bonyeza G - siku 7 Bonyeza H | |
| 1. Katika siku 7 zilizopita,ni mara ngapi umetembea au kupeleka baiskeli kwenda au kutoka shule? | - kama ni siku 0/bado BonyezaA - kama ni kwa siku 1 Bonyeza B - kama ni kwa siku 2 Bonyeza C - kama ni kwa siku 3 Bonyeza D - kama ni kwa siku 4 Bonyeza E - kama ni kwa siku 5 Bonyeza F - kama ni kwa siku 6 Bonyeza G - kama ni kwa siku 7 Bonyeza H | |
| 1. Katika mwaka huu wa shule,ni siku ngapi umeenda darasa la elimu ya mazoezi ya mwili kila wiki? | - siku 0/bado Bonyeza A - siku 1 Bonyeza B - siku 2 Bonyeza C - siku 3 Bonyeza D - siku 4 Bonyeza E - siku 5 au zaidi Bonyeza F | |
| **Swali lifuatalo ni kuhusu wakati unaotumia sana kuketi kama huko shule au kufanya homework.(kwa watoto wanaoenda shule)** | | |
| 1. Ni muda gani unatumia kwa siku ya kawaida,kuketi na kuangalia runinga,kuongea na marafiki au kufanya shughuli za kuketi kama vile kutoa hadithi. | - Kama ni chini ya saa 1 kwa siku Bonyeza A - Kama ni kwa saa 1 hadi 2 kwa siku Bonyeza B - Kama ni kwa saa 3 hadi 4 kwa siku Bonyeza C - Kama ni kwa saa 5 hadi 6 kwa siku Bonyeza D - Kama ni kwa saa 7 hadi 8 kwa siku Bonyeza E - Kama ni kwa zaidi ya masaa 8 kwa siku Bonyeza F | |
| **Maswali 6 yafuatayo yanauliza kuhusu uzoefu shule na nyumbani** | | |
| 1. Katika siku 30 zilizopita,ni siku ngapi ulikosa darasa au shule bila ruhusa? | - Kama ni kwa siku 0/bado Bonyeza A - Kama ni kwa siku 1 au 2 Bonyeza B - Kama ni kwa siku 3 hadi 5 Bonyeza C - Kama ni kwa siku 6 hadi 9 Bonyeza D - Kama ni kwa siku 10 au zaidi Bonyeza E | |
| 1. Katika siku 30 zilizopita,ni mara ngapi wanafunzi wengi shuleni kwenu walikuwa wakarimu na wa msaada. | - kama ni hapana Bonyeza A - kama si sana/nadra Bonyeza B - kama ni mara nyingine Bonyeza C - kama ni kwa mara nyingi Bonyeza D - kama ni kila wakati Bonyeza E | |
| 1. Katika siku 30 zilizopita,ni mara ngapi wazazi/walezi wako waliangalia kama kazi ya shule imefanywa? | - kama ni hapana Bonyeza A - kama si sana/nadra Bonyeza B - kama ni mara nyingine Bonyeza C - kama ni kwa mara nyingi Bonyeza D - kama ni kila wakati Bonyeza E | |
| 1. Katika siku 30 zilizopita,ni mara ngapi wazazi/walezi walikuelewa shida na wasiwasi wako? | - kama ni hapana Bonyeza A - kama si sana/nadra Bonyeza B - kama ni mara nyingine Bonyeza C - kama ni kwa mara nyingi Bonyeza D - kama ni kila wakati Bonyeza E | |
| 1. Katika siku 30 zilizopita, ni mara ngapi wazazi /walezi wako wamejua kwa hakika unalofanya wakati uko huru? | - kama ni hapana Bonyeza A - kama si sana/nadra Bonyeza B - kama ni mara nyingine Bonyeza C - kama ni kwa mara nyingi Bonyeza D - kama ni kila wakati Bonyeza E | |
| 1. Katika siku 30 zilizopita,ni mara ngapi wazazi/walezi wako wameangalia vitu vyako bila idhini yako? | - kama ni hapana Bonyeza A - kama si sana/nadra Bonyeza B - kama ni mara nyingine Bonyeza C - kama ni kwa mara nyingi Bonyeza D - kama ni kila wakati Bonyeza E | |
| **Maswali 6 yafuatayo yanauliza kuhusu sigara na matumizi mengine ya tumbaku** | | |
| 1. Je ulikuwa na umri gani ulipojaribu kuvuta sigara? | - Kama hujawahi kuvuta sigara Bonyeza A - Kama ni miaka 7 au chini yake Bonyeza B - Kama ni miaka 8 au 9 Bonyeza C - Kama ni miaka 10 au 11 Bonyeza D - Kama ni miaka 12 au 13 Bonyeza E - Kama ni miaka 14 au 15 Bonyeza F - Kama ni miaka 16 au17 Bonyeza G - Kama ni miaka 18 au zaidi Bonyeza H | |
| 1. Katika siku 30 zilizopita,ni siku ngapi ulivuta sigara? | - kama ni siku 0/bado Bonyeza A - kama ni siku 1 au 2 Bonyeza B - kama ni siku 3 hadi 5 Bonyeza C - kama ni siku 6 hadi 9 Bonyeza D - kama ni siku 10 hadi 19 Bonyeza E - kama ni siku 20 hadi 29 Bonyeza F - siku zote 30 Bonyeza G | |
| 1. Katika siku 30 zilizopita,ni siku ngapi ulitumia bidhaa yoyote ya tumbaku mbali na sigara, kama kiko? | - kama ni siku 0/bado Bonyeza A - kama ni siku 1 au 2 Bonyeza B - kama ni siku 3 hadi 5 Bonyeza C - kama ni siku 6 hadi 9 Bonyeza D - kama ni siku 10 hadi 19 Bonyeza E - kama ni siku 20 hadi 29 Bonyeza F - kama ni siku zote 30 Bonyeza G | |
| 1. Katika miezi 12 iliyopita,je umejaribu kuacha kuvuta sigara? | - Kama hujawahi kuvuta sigara Bonyeza A - kama hukuvuta sigara katika miezi 12 iliyopita Bonyeza B - kama ni ndio Bonyeza C - kama ni la Bonyeza D | |
| 1. Katika siku 7 zilizopita,ni siku ngapi watu wamevuta mbele yako? | - siku 0 - siku 1 au 2 - siku 3 au 4 - mara 5 au 6 - siku zote 7 | |
| 1. Ni mzazi/mlezi yupi hutumia aina yoyote ya tumbaku? | - hakuna Bonyeza A - kama ni babako au mlezi wako wa kiume Bonyeza B - kama ni mamako au mlezi wako wa kiume Bonyeza C - wote Bonyeza D - hujui Bonyeza E | |
| **Swali lifuatalo ni kuhusu kupigana vita.Kupigana vita hutokea ikiwa wanafunzi wawili wa nguvu au mamlaka sawa wanaamua kupigana.** | | |
| 1. Katika miezi 12 iliyopita, ni mara ngapi ulipigana vita? | - kama ni mara 0/bado Bonyeza A - kama ni mara 1 Bonyeza B - kama ni mara 2 au 3 Bonyeza C - kama ni mara 4 au 5 Bonyeza D - kama ni mara 6 au 7 Bonyeza E - kama ni mara 8 au 9 Bonyeza F - kama ni mara 10 au 11 Bonyeza G - kama ni mara 12 au zaidi Bonyeza H | |
| **Maswali 3 yafuatayo yanauliza majeraha mabaya yaliyokupata.jeraha ni baya kama litakufanya ukose angalau moja ya shughuli zako za kawaida (kama shule,michezo au kazini) au inahitaji matibabu ya daktari au muuguzi.** | | |
| 1. Katika miezi 12 iliyopitani mara ngapi ulijeruhiwa vibaya? | - kama ni mara 0/bado Bonyeza A - kama ni mara 1 Bonyeza B - kama ni mara 2 au 3 Bonyeza C - kama ni mara 4 au 5 Bonyeza D - kama ni mara 6 au 7 Bonyeza E - kama ni mara 8 au 9 Bonyeza F - kama ni mara 10 au 11 Bonyeza G - kama ni mara 12 au zaidi Bonyeza H | |
| 1. Katika miezi 12 iliyopita,ni jeraha gani mbaya zaidi ulilolipata? | - Kama hukujeruhiwa vibaya katika miezi 12 iliyopita Bonyeza A - Kama hukuvunjika mfupa au kuteguka Bonyeza B . - Kama ulikatika au ulikuwa na kidonda cha kupigwa Bonyeza C - Kama ulipata mtikiso au jeraha la kichwa au shingo,ulishindwa na usingeweza vuta pumzi - Kama ulipatalipata jeraha la risasi Bonyeza E - Kama ulichomeka Bonyeza F - Kama ulitiliwa sumu au ulitumia dawa zaidi Bonyeza G - Kama jambo lengine lilikupata Bonyeza H | |
| 1. Katika miezi 12 iliyopita,nini kilisababisha jeraha hilo baya lililokupata.? | - Kama hukujeruhiwa vibaya katika katika miezi 12 iliyopita Bonyeza A - Kama hukuhusika na ajali ya gari au uligongwa na gari Bonyeza B - Kama ulianguka Bonyeza C - Kama uliangukiwa na kitu au uligogwa Bonyeza D - Kama ulivamiwa au kudhihakiwa/kudhulumiwa au ulipigana na mtu Bonyeza E - Kama ulikuwa kwenye moto au karibu na miale au kitu cha moto Bonyeza F - Kama ulivuta au meza kitu kibaya kwako Bonyeza G - Kama ni Jambo lengine lilisababisha jeraha Bonyeza H | |
| **Maswali 2 yafuatayo ni kuhusu kuteswa/kusumbuliwa.Hii inatokea wakati mwanafunzi au kikundi cha wanafunzi husema au kufanya vitu vibaya kwa mwanafunzi mwengine.Pia ni kusumbuliwa wakati mwanafunzi anachezewa sana kwa njia isiyofurahisha au wakati mwanafunzi anaachwa kando ya vitu kwa makusudi.Si kuteswa/kusumbuliwa wakati wanafunzi wawili wa nguvu na mamlaka sawa wanapojadili au kuteta au kama kuchezeana ni kwa kirafiki na kufurahisha.** | | |
| 1. Katika siku 30 zilizopita,ni siku ngapi uliteswa? | - Kama ni mara 0/hukuteswa Bonyeza A - kama ni mara 1 au 2 Bonyeza B - kama ni mara 3 hadi 5 Bonyeza C - kama ni mara 6 hadi 9 Bonyeza D - kama ni mara 10 hadi 19 Bonyeza E - kama ni mara 20 hadi 29 Bonyeza F - kama ni siku zote 30 Bonyeza G | |
| 1. Katika siku 30 zilizopita,uliteswa/sumbuliwa vipi mara nyingi? | - Kama hukuteswa/kusumbuliwa katika muda wa siku 30 zilizopita Bonyeza A - Kama uligongwa,ukasukumwa sukumwa au kufungiwa ndani Bonyeza B - Kama ulichekwa kwa sababu ya uraia au rangi Bonyeza C - Kama ulichekwa kwa sababu ya dini yako Bonyeza D - Kama ulichekwa na matani ya ngono,maneno,au ishara Bonyeza E - Kama ulitengwa mbali na shuhuli kimakusudi au kutoshuhulikiwa kabisa Bonyeza F. - Kama ulichekwa kwa sababu ya mwili au uso vile unaonekana Bonyeza G . - Kama uliteswa kwa njia nyengine Bonyeza H | |
| **Maswali mawili yafuatayo yanahusu ndoa** | | |
| 1. Je hali yako ya ndoa ikoje? | - Kama umeolewa/umeoa Bonyeza A - Kama hamjaoana lakini unaishi na mwenzio wa jinsia tofauti Bonyeza B - Kama umefiwa Bonyeza C - Kama mmeachana Bonyeza D - Kama mmetengana kwa sababu wewe na mke/mume wako hamuelewani Bonyeza E - Kama hujaolewa/hujaoa Bonyeza F | |
| 1. Je ulikuwa na umri gani wakati wa ndoa yako ya kwanza? | - Kama ni chini ya au miaka 13 Bonyeza A - Kama ni miaka 14 Bonyeza B - Kama ni miaka 15 Bonyeza C - Kama ni miaka 18 Bonyeza D - Kama ni miaka 17 Bonyeza E - Kama ni miaka 18 au zaidi Bonyeza F - Kama hujui Bonyeza G | |
| **Pia, ningetaka kuongea kuhusu uja uzito.** | | |
| 1. Ni mara ngapi umepata uja uzito au kupatia mtu uja uzito? | - Kama ni mara 0/bado Bonyeza A - Kama ni mara 1Bonyeza B - Kama ni mara mbili au zaidi Bonyeza C - Kama hujui Bonyeza D | |
| 1. Je ulikuwa na umri gani ulipopata mimba yako ya kwanza?(wanawake pekee) | - Kama ni chini ya au miaka 13 Bonyeza A - Kama ni miaka 14 Bonyeza B - Kama ni miaka 15 Bonyeza C - Kama ni miaka 16 Bonyeza D - Kama ni miaka 17 Bonyeza E - Kama ni miaka 18 au zaidi Bonyeza F - Kama hujui Bonyeza G | |
| 1. Je matokeo ya uja uzito yalikuwaje? | - Kama ulijifugua mtoto hai Bonyeza A - Kama bado mjamzito Bonyeza B - Kama ulijifungua mtoto wa kufa Bonyeza C - Kama uliavya Bonyeza D - Kama hujui Bonyeza E | |

| **Sehemu hii ya mwisho ni kuhusu tabia za ngono** | |
| --- | --- |
| 1. Je umewahi kushiriki ngono? | - Kama ni ndio Bonyeza A - Kama ni La Bonyeza B |
| 1. Je ulikuwa na umri gani uliposhiriki ngono mara ya kwanza? | - Kama hujawahi kushiriki ngono Bonyeza A - Kama ni miaka 11 au chini yake Bonyeza B - Kama ni miaka 12 Bonyeza C - Kama ni miaka 13 Bonyeza D - Kama ni miaka 14 Bonyeza E - Kama ni miaka 15 Bonyeza F - Kama ni miaka 16 au 17 Bonyeza G - Kama ni miaka 18 au zaidi Bonyeza H |
| 1. Katika maisha yako, ni watu wangapi umeshiriki nao ngono? | - Kama hujawahi kushiriki ngono Bonyeza A - Kama ni mtu 1 Bonyeza B - Kama ni watu 2 Bonyeza C - Kama ni watu 3 Bonyeza D - Kama ni watu 4 Bonyeza E - Kama ni watu 5 Bonyeza D - Kama ni watu 6 au zaidi Bonyeza G |
| 1. Mara ya mwisho uliposhiriki ngono, je wewe au mwenzi wako mlitumia mpira? | - Kama hujawahi kushiriki ngono Bonyeza A - Kama ndio Bonyeza B - Kama La Bonyeza C |
| 1. Mara ya mwisho mliposhiriki ngono,je wewe au mwenzi wako mlitumia njia nyengine yoyote ya kupanga uzazi kama kujitoa,wakati unaofaa,tembe ya kuzuia kuzaa au njia nyengine ya kuzuia mimba? | - Kama hujawahi kushiriki ngono Bonyeza A - Kama ni ndio Bonyeza B - Kama ni la Bonyeza C - Kama hujui Bonyeza D |
| 1. Je ushawahi kudanganywa kufanya ngono bila wewe kutaka? | - kama ni ndio Bonyeza A - kama ni la Bonyeza B |
| 1. Je umeshawahi kufungiwa chumbani ili kufanya ngono,bila kutaka? | - kama ni ndio Bonyeza A - kama ni la Bonyeza B |
| 1. Je ushawahi kulazimishwa kwa nguvu kufanya ngono bila kutaka? | - Kama ni ndio Bonyeza A - Kama ni la Bonyeza B |
| 1. Ni wapi ulitafuta/ulipata usaidizi? | - Kama hukutafuta usaidizi Bonyeza A - Kama Kituo cha afya Bonyeza B - Kama ni polisi Bonyeza C - Kama kituo cha mtaa/baraza la mtaa Bonyeza D - Kama ni kwa haki za mtoto Bonyeza E - Kama sii moja kati yaliyoorodheshwa Bonyeza F. |
